# Broad spectrum immunomodulatory effects of *Anopheles gambiae* microRNAs and their use for transgenic suppression of *Plasmodium*

**DOI:** 10.1371/journal.ppat.1008453

**Published:** 2020-04-24

**Authors:** Shengzhang Dong, Xiaonan Fu, Yuemei Dong, Maria L. Simões, Jinsong Zhu, George Dimopoulos

**Affiliations:** 1 W. Harry Feinstone Department of Molecular Microbiology and Immunology, Bloomberg School of Public Health, Johns Hopkins University, Baltimore, MD, United States of America; 2 The Interdisciplinary Ph.D. Program in Genetics, Bioinformatics, and Computational Biology, Virginia Tech, Blacksburg, VA, United States of America; 3 Department of Biochemistry, Virginia Tech, Blacksburg, VA, United States of America; University of California, Riverside, UNITED STATES

## Abstract

Malaria, caused by the protozoan parasite *Plasmodium* and transmitted by *Anopheles* mosquitoes, represents a major threat to human health. *Plasmodium*’s infection cycle in the *Anopheles* vector is critical for transmission of the parasite between humans. The midgut-stage bottleneck of infection is largely imposed by the mosquito’s innate immune system. microRNAs (miRNAs, small noncoding RNAs that bind to target RNAs to regulate gene expression) are also involved in regulating immunity and the anti-*Plasmodium* defense in mosquitoes. Here, we characterized the mosquito’s miRNA responses to *Plasmodium* infection using an improved crosslinking and immunoprecipitation (CLIP) method, termed covalent ligation of endogenous Argonaute-bound RNAs (CLEAR)-CLIP. Three candidate miRNAs’ influence on *P*. *falciparum* infection and midgut microbiota was studied through transgenically expressed miRNA sponges (miR-SPs) in midgut and fat body tissues. MiR-SPs mediated conditional depletion of aga-miR-14 or aga-miR-305, but not aga-miR-8, increased mosquito resistance to both *P*. *falciparum* and *P*. *berghei* infection, and enhanced the mosquitoes’ antibacterial defenses. Transcriptome analysis revealed that depletion of aga-miR-14 or aga-miR-305 resulted in an increased expression of multiple immunity-related and anti-*Plasmodium* genes in mosquito midguts. The overall fitness cost of conditionally expressed miR-SPs was low, with only one of eight fitness parameters being adversely affected. Taken together, our results demonstrate that targeting mosquito miRNA by conditional expression of miR-SPs may have potential for the development of malaria control through genetically engineered mosquitoes.

## Author summary

Malaria is caused by the *Plasmodium* parasite that is transmitted by *Anopheles* mosquitoes. The mosquito’s innate immune system plays an important role in controlling parasite infection. We have identified mosquito microRNAs (miRNAs) that are involved in regulating mosquito immunity to parasite infection. Transgenic mosquitoes that deplete the immunity-related miRNAs aga-miR-14 or aga-miR-305 through miRNA sponges, show increased resistance to both human and rodent parasite infection, and enhanced antibacterial defenses. Depletion of aga-miR-14 or aga-miR-305 resulted in an increased expression of multiple immunity-related and anti-*Plasmodium* genes, and the overall fitness cost of transgenic mosquitoes upon depletion of aga-miR-14 or aga-miR-305 was negligible. We show that targeting mosquito miRNA by transgenic expression of miRNA sponges may have potential for the development of malaria control through genetically engineered mosquitoes.

## Introduction

Malaria is caused by protozoan parasites belonging to the genus *Plasmodium* that are transmitted by female *Anopheles* mosquitoes; this disease was responsible for up to half a million deaths worldwide in 2017 [1]. Transmission of *Plasmodium* relies on the successful completion of its complex lifecycle in *Anopheles* mosquitoes. After ingestion during blood feeding, malaria parasites undergo sexual reproduction inside the midgut lumen of a female *Anopheles* mosquito and differentiate into motile ookinetes. After traversing the midgut epithelium, the ookinetes transform into oocysts, within which thousands of sporozoites develop and are released to invade the salivary glands. From there they can be transmitted to humans during subsequent bites [2]. The major bottleneck for malaria parasite development in mosquitoes occurs during developmental transitions in the midgut lumen and during ookinete invasion of the midgut epithelium, prior to the development of oocysts on the basal lamina [3]. This bottleneck is to a significant degree imposed by the mosquito’s innate immune system, which is largely guided by the immune deficiency (Imd), Toll, Janus kinase/signal transducers and activators of transcription (JAK/STAT), and c-Jun N-terminal kinase (JNK) pathways [4, 5]. Overexpression of the Imd pathway transcription factor (*Rel2*) in midguts of transgenic *Anopheles stephensi* mosquitoes significantly reduces parasite oocyst numbers in the midgut [6], demonstrating that transgenic enhancement of mosquito immunity may provide an effective approach for controlling the spread of malaria [7].

MicroRNAs (miRNAs) are small endogenous noncoding RNAs that regulate gene expression, primarily via RNA decay or posttranslational inhibition [8]. Most mosquito miRNA studies have concentrated on identification and annotation using high-throughput sequencing and subsequent bioinformatics analysis [9–14]. However, only a few miRNAs have been functionally studied in malaria mosquitoes [15, 16] partly because of the lack of genome-wide resources for assessing loss of miRNA function. Recently, miRNA sponges (miR-SPs) were successfully used to define the functions of specific miRNAs in multiple species and biological contexts [17]. MiR-SPs contain multiple or “bulged” tandem target sites for binding to a miRNA of interest. MiR-SPs can be transgenically expressed by specific promoters to sequester target miRNAs and thus influence the function of miRNA-targeted genes [18].

The miRNA biosynthetic pathway, and specific miRNAs, have been shown to influence anti-*Plasmodium* immunity in *Anopheles*, and the expression of multiple miRNAs is modulated by *Plasmodium* infection [14, 15]. Several miRNAs have also been predicted *in silico* to regulate the expression of immune genes and anti-*Plasmodium* effectors. However, a high-throughput method was required to identify which of the 168 miRNAs actually influence mosquito immunity. High-throughput sequencing of RNAs isolated by crosslinking and immunoprecipitation (CLIP) of Argonaute has been applied to generate functional miRNA-mRNA interaction maps in developing *Caenorhabditis elegans* and in mouse brain [19, 20]. An improved CLIP method, termed covalent ligation of endogenous Argonaute-bound RNAs (CLEAR)-CLIP, has an additional ligation step to join the miRNA and its targeted mRNA in the purified miRISC complex into one chimeric molecule [21]. Analysis of the chimeric miRNA-target molecules in RNAs associated with Ago allows for systematic identification of miRNA-target interactions [22, 23].

Here, we applied CLEAR-CLIP to uncover *An*. *gambiae* miRNA-target interactions that were affected by *Plasmodium* infection, and screen for miRNAs with potential function in anti-*Plasmodium* immunity. In our previous work we have used a straight-forward cost- and time-effective antagomir-based miRNA silencing approach to assess whether selected miRNAs are implicated in *Plasmodium* suppression. Here we developed and used a transgenic approach to target immunity-regulating miRNAs through conditional expression of miR-SPs in the midgut or fat body, in order to investigate their potential involvement in regulating *Plasmodium* infection, as well as to examine the fitness impact of their targeted miRNA depletion. Advantages of the less invasive transgenic approach are that it enables endogenous miRNA depletion in a tissue-specific manner, the study of the impact on the midgut transcriptome as well as fitness parameters. It also provides an evaluation of the strategy for the development of *Plasmodium* resistant mosquitoes. Transgenic depletion of immunity-regulating miRNAs up-regulated numerous immunity and anti-*Plasmodium* genes, increased mosquito refractoriness to *Plasmodium* infection, suppressed the midgut microbiota, and only reduced the longevity of blood-fed transgenic females without affecting other fitness parameters.

## Results

### CLEAR-CLIP defines miRNA-mRNA interactions during *Plasmodium* infection in *An*. *gambiae*

**Fig 1:**
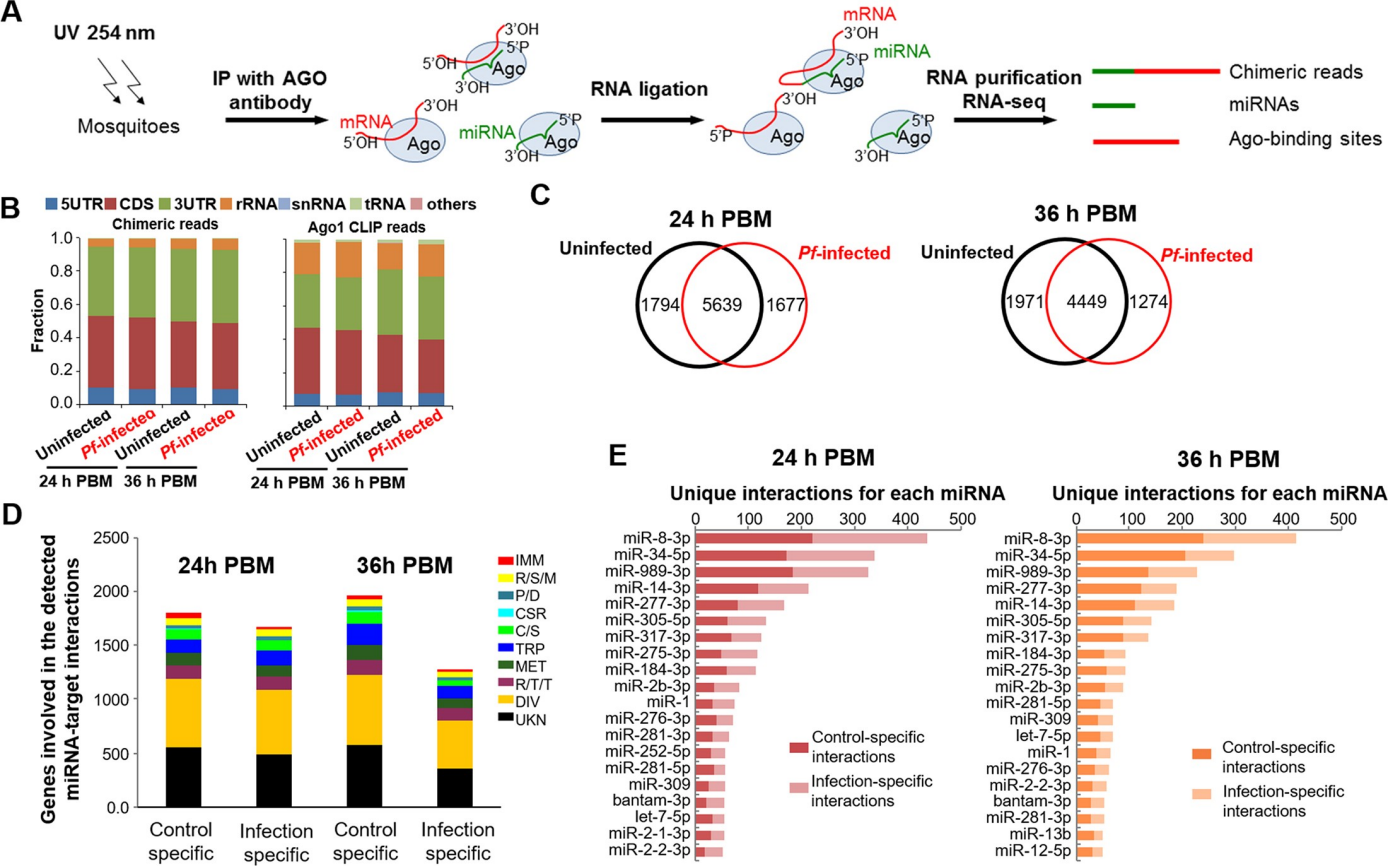
*Plasmodium* infection substantially affects the miRNA-mRNA interactions in mosquitoes. (**A**) Overview of the CLEAR-CLIP experimental procedure. (**B**) Mapping of standard Ago1 CLIP reads and miRNA-target chimeras to mosquito transcriptomes. (**C**) Venn diagram showing the differential miRNA-mRNA interactions between uninfected and infected mosquitoes at 24 h PBM and 36 h PBM. (**D**) Number of mosquito genes from known functional categories that display altered interaction with miRNAs in response to *Plasmodium* infection. C/S, cytoskeletal and structural; CSR, chemosensory reception; DIV, diverse functions; IMM, immunity; MET, metabolism; PRT, proteolysis; R/S/M, redox, stress, and mitochondrion; R/T/T, replication, transcription, and translation; TRP, transport; UKN, unknown functions. (E) Most frequently found miRNAs being involved in the differential miRNA-mRNA interactions in response to *Plasmodium* infection. Only the mutually exclusive infection-specific or control-specific interactions were included for this analysis.

*An*. *gambiae* fed with either uninfected or *P*. *falciparum*-infected (*Pf*-infected) blood were subjected to CLEAR-CLIP analysis at 24 h and 36 h post blood meal (PBM) when *Plasmodium* ookinetes invade the midgut epithelium (**Fig 1A**). Mosquito whole bodies were collected at 24 h and 36 h PBM. A total of 213,940,116 reads were obtained from 12 CLEAR-CLIP libraries (**S1 Table**), comprising miRNA-mRNA chimeras (<3% of mapped unique reads), mRNA “single reads,” miRNA “single reads,” and other noncoding RNAs. Clustering of the mRNA single reads (hereafter referred to as Ago1 CLIP reads) generated enriched clusters that represented the binding sites of Ago1. MiRNA targets derived from the chimeric reads were mostly located in the 3’-untranslated regions (3’UTR; 41.67%) and coding regions (CDS; 41.88%) of mosquito genes, but were also present in the 5’-untranslated region (5’UTR; 9.72%) (**Fig 1B**). This distribution remained largely unchanged between infected and uninfected mosquitoes (**Fig 1**).

There are several lines of evidence indicating that the chimeras resulted primarily from the miRNA-mRNA interactions in the miRISC. First, about 70% of the mRNA sequences in the chimeras were also recovered as a cluster of single reads in the same library (**S1 Fig**). Second, the mean predicted free energy between the miRNAs and matching target mRNAs found in the chimeras was 4.4 kcal mol^-1^ lower than that in randomly matched pairs (*p*<0.001). Third, enriched motifs were found in the CLEAR-derived targets of many miRNAs. The motifs were mostly complementary to the miRNA seed regions (**S2 Fig**). Fourth, some well-characterized miRNA-target interactions were captured in the CLEAR-CLIP experiments, including miR-309-*SIX4* and miR-8-*Swim*, which were first reported in *Ae*. *aegypti* [24, 25] (**S1 Fig**).

### *Plasmodium* infection affects the miRNA-mRNA interactions in *An*. *gambiae*

To remove chimeras derived from random ligation, only chimeras that were mapped to Ago1 CLIP peaks were retained for subsequent analysis. If specific chimeras were detected in the *Pf*-infected mosquitoes but not in the control groups, the absence of the chimeras might be attributed to the low efficiency in the ligation of miRNA and mRNA pairs in the CLEAR experiment. Therefore, when we assessed whether a specific interaction was affected by *Plasmodium* infection, both chimeras and corresponding Ago 1 CLIP peaks were taken into consideration. Overall, 9041 unique miRNA-target pairs were mapped (**S2 Table**). The miRNA-target interactome in *An*. *gambiae* was clearly altered in response to malaria parasite infection (**Fig 1C**). Genes differentially regulated by miRNAs during *Plasmodium* infection covered a wide range of functional categories, from metabolism to immunity (**Fig 1D**). The altered miRNA-target interactome thus suggested that the miRNA-mRNA interactions play important roles in the immune and other physiological responses to *Plasmodium* infection.

Aga-miR-8-3p, aga-miR-34-5p, aga-miR-989-3p, aga-miR-14-3p, aga-miR-277-3p, and aga-miR-305-5p were the top six miRNAs that were involved in the differential miRNA-target interactions at both 24 h PBM and 36 h PBM (**Fig 1E**). We selected three miRNAs (aga-miR-8, aga-miR-14, and aga-miR-305) for the functional study based on the following criteria: miR-8 was the most abundant miRNA candidate identified using CLEAR-CLIP approach, miR-14 was predicted, through our previous study and current CLEAR-CLIP analysis, to target multiple immune genes, and antagomir-mediated inhibition of miR-305 increased mosquito resistance to *P*. *falciparum* infection, and miR-305 was also shown to be involved in the differential miRNA-target interactions [15].

### MiRNAs are depleted by miRNA sponges-overexpressing transgenic mosquitoes

**Fig 2:**
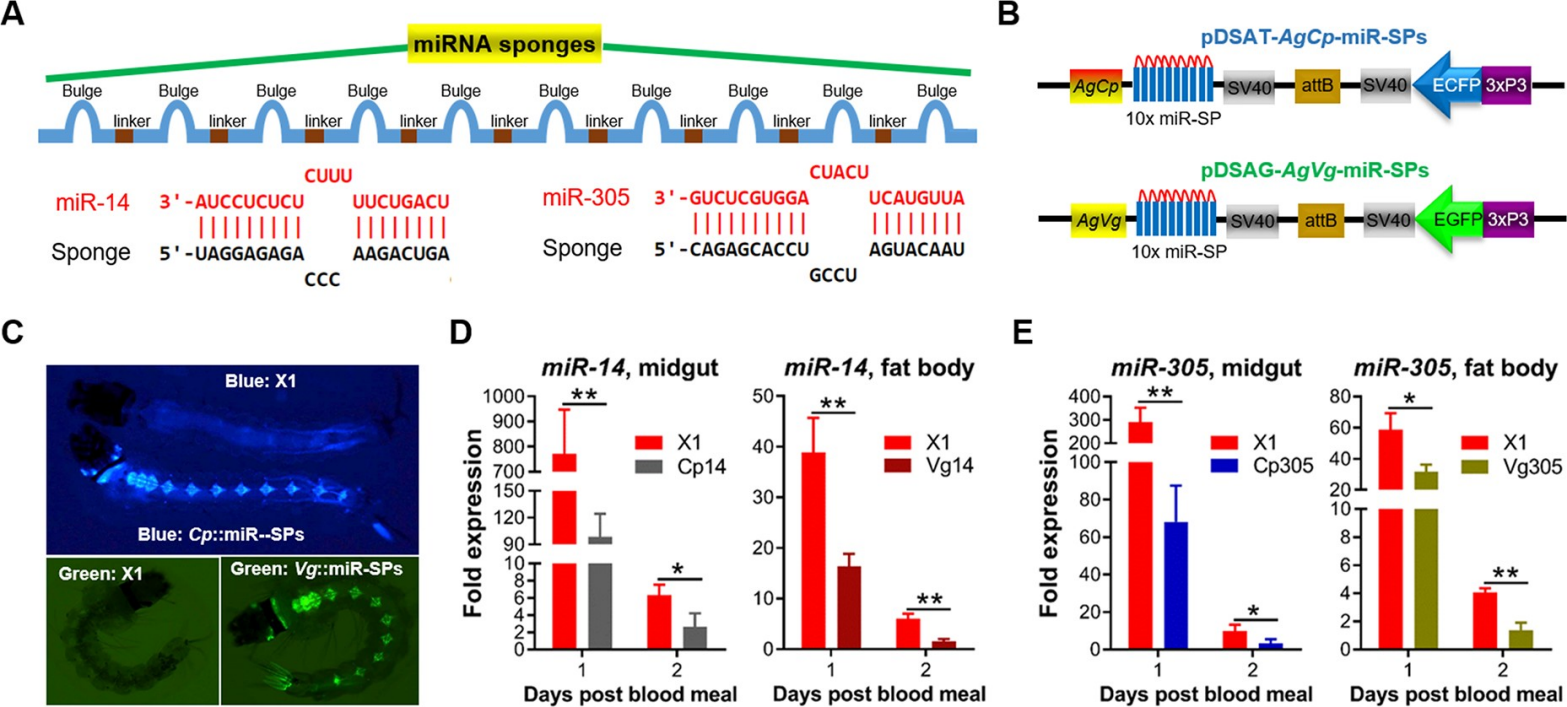
Generation and characterization of aga-miR-14 and aga-miR-305 sponges (SPs) overexpressing transgenic *An*. *gambiae*. (**A**) miRNA-SPs with 10 repetitive aga-miR-14 (or aga-miR-305) complementary sequences separated by variable four-nucleotide linker sequences and a four-nucleotide bulge at the central MBS (10x miR-SPs). (**B**) Scheme of pDSA constructs for overexpressing microRNA-SPs under the control of the blood-induced midgut-specific promoter (*AgCp*) or fat body-specific promoter (*AgVg*). Abbreviations: *AgCp*, *An*. *gambiae carboxypeptidase* promoter; *AgVg*, *An*. *gambiae vitellogenin* promoter; sv40, polyadenylation signal of simian virus 40 VP1 gene; 3xP3, eye tissue-specific promoter; attB, phage Φ31 attB site. (**C**) *AgCp* control of the miR-SPs transgenic line with blue fluorescence marker, and the *AgVg* line with green fluorescence marker. The docking line (X1) has no fluorescence in the eyes. Expression of aga-miR-14 (**D**) and aga-miR-305 (**E**) was significantly decreased in the midguts or fat bodies of miR-SPs transgenic mosquitoes following a blood meal. MicroRNA expression was detected by qPCR and normalized to rS7. Cp14 (305) and Vg14 (305): aga-miR-14 (305) sponges overexpressed in midguts in response to the *Cp* promoter or in fat bodies in response to the *Vg* promoter. Statistical comparisons of miRNA expression between transgenic lines and the docking line (X1) were performed using the unpaired *t*-test and error bars represented the standard deviation (SD) of the mean. *P<0.05, **P<0.01.

To deplete specific miRNAs, we synthesized miR-SPs consisting of 10 or 20 tandem miRNA-binding sites (MBS), each separated by a four-nucleotide spacer sequence (**Fig 2A and S3 Fig**) and introduced a four-nucleotide bulge at the central MBS to make sponges more stable and effective. The miR-SPs cassette was cloned into the pDSAT/G plasmid with either the *carboxypeptidase* (*Cp*) or *vitellogenin* (*Vg*) promoter to enable midgut- or fat body-specific expression following a blood meal (**Fig 2B and S3A Fig**) [6, 26]. Our previous studies have shown that immune responses both in the midgut and fat body tissues can affect *Plasmodium* infection in the midgut [6], and for this reason we used both *Cp* and *Vg* promoters. Plasmid constructs were microinjected together with a helper plasmid, which expresses Φ31 integrase under the control of germline promoter *Vasa*, into the embryos of the *An*. *gambiae* docking line X1 [27]. Each injection involved 500 to 1100 embryos, and the G0 survival rate ranged from 10 to 20% (**S3 Table**). First instar larvae of G0 survivors were screened for transient fluorescence, and the positive G0 adults were backcrossed to the parental X1 line. G1-positive mosquitoes were either incrossed or outcrossed with X1 mosquitoes to expand the colony.

A total of six transgenic lines were generated: two for each miRNA driven by either the *Cp* or *Vg* promoter (**Fig 2 and S3 Table**), denoted Cp14, Vg14, Cp305, Vg305, Cp8, and Vg8 (**S4 Fig**). All six transgenic lines showed strong adult eye fluorescence and strong larval and adult nervous system fluorescence (**Fig 2C and S3C and S4 Figs**). To confirm the efficiency of the targeted miRNA depletion, qPCR was used to assay the abundance of the target miRNA in transgenic mosquitoes. The results showed a significant depletion of miRNAs in the midguts of all *Cp* lines and fat bodies of all *Vg* lines on days 1 and 2 PBM when compared to the docking line X1 (**Fig 2D and 2E and S3D Fig**). All three *Cp* lines showed a strong depletion efficiency at 24 h PBM, in contrast to at 2 days PBM in all three *Vg* lines, consistent with the timing of *Cp* or *Vg* promoter activation by the blood meal [6, 26].

### Sponges-mediated depletion of aga-miR-14 or aga-miR-305 enhances mosquito resistance to *Plasmodium* infection

**Fig 3:**
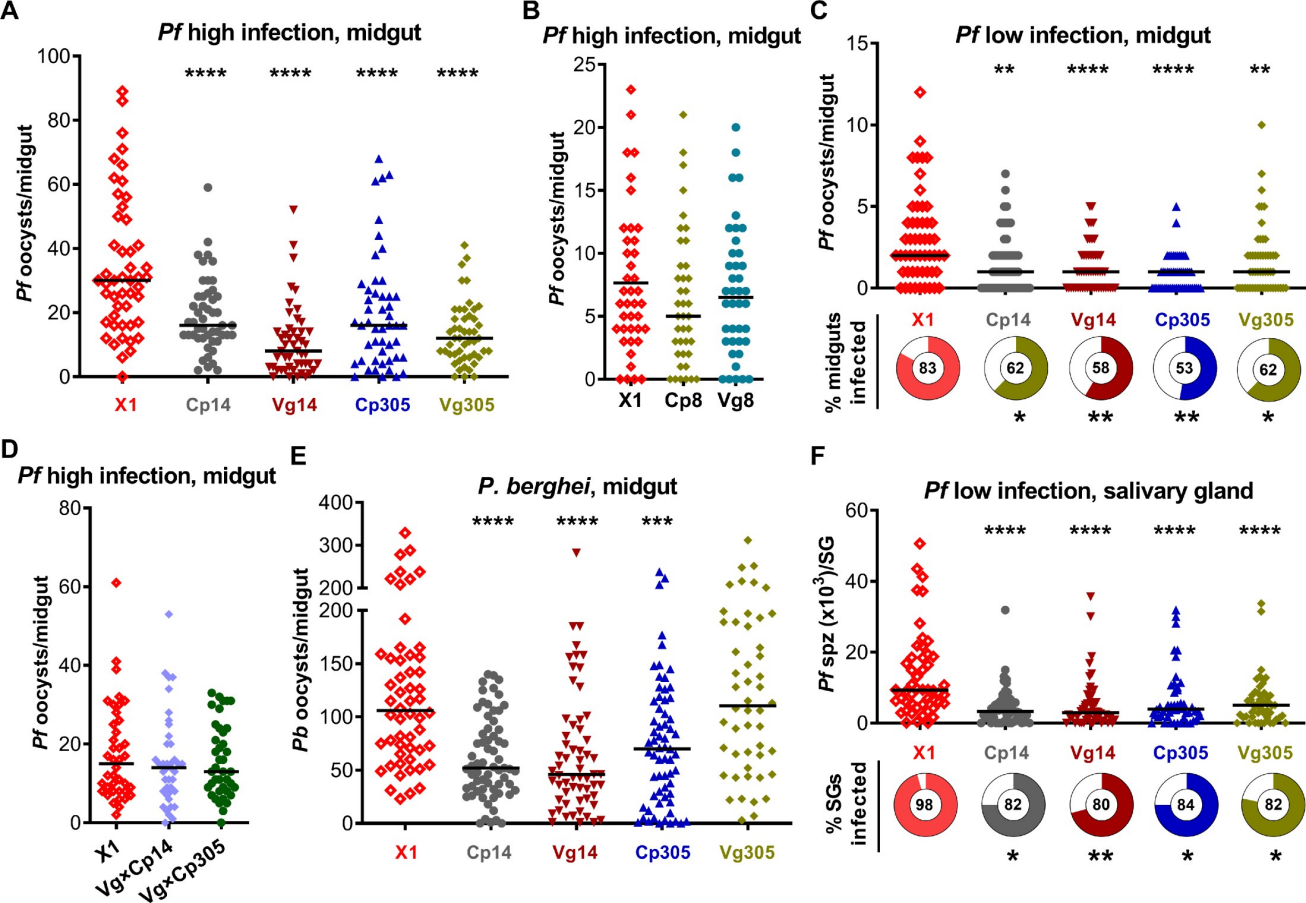
Sponges-mediated depletion of aga-miR-305 or aga-miR-14, but not aga-miR-8, results in *Plasmodium* suppression in transgenic *An*. *gambiae*. (**A**) *P*. *falciparum* (*Pf*) oocyst number in midguts of transgenic lines (miR-14 and miR-305) and the docking line (X1) 8 days after feeding on high gametocytaemic blood. (**B**) *P*. *falciparum* oocyst number in midguts of transgenic lines (miR-8) and X1 at 8 days after feeding on high gametocytaemic blood. (**C**) *P*. *falciparum* oocyst number and infection prevalence in midguts of transgenic lines and X1 8 days after feeding on low gametocytaemic blood. (**D**) *P*. *falciparum* oocyst number in midguts of hybrids of Cp14×Vg14 or Cp305×Vg305 and X1 at 8 days after feeding on high gametocytaemic blood. (**E**) *P*. *berghei* (*Pb*) oocyst number in midguts of transgenic lines and X1 at 13 days post-infection when fed on *P*. *berghei*-infected mice. (**F**) *P*. *falciparum* sporozoite number and infection prevalence in salivary glands of transgenic lines and X1 14 days after feeding on low gametocytaemic blood. Points indicate the absolute value of oocyst counts in individual midguts or sporozoite counts in individual salivary glands, and horizontal black bars in each column represent the median value of the oocyst or sporozoite number. The donut charts and the number in the middle of each donut chart indicate the percentage of midguts or salivary glands infected by parasites. Assays were performed with three biological replicates (**A** and **F**) or two replicates (**B**-**E**), and equal numbers of midguts or salivary glands from different replicates were pooled for the dot-plots. Statistical comparisons of oocyst or sporozoite numbers and infection prevalence between transgenic lines and X1 were performed using the Mann-Whitney test or Fisher's exact test, respectively. *P<0.05, **P<0.01, ***P<0.001, ****P<0.0001.

To determine the effect of the miRNA depletion on *P*. *falciparum* infection, we fed a pool of homozygous or heterozygous transgenic hybrids (*Cp*×*Vg*), along with docking line X1 mosquitoes as a control, on an NF54 *P*. *falciparum* gametocyte culture. When mosquitoes were infected through a highly gametocytaemic culture, depletion of miR-14 or miR-305, but not miR-8, in the midgut or fat body resulted in a significantly decreased median number of oocysts when compared to X1 mosquitoes (**Fig 3A and 3B**). Therefore, we selected the four miR-14 and miR-305 sponges-expressing homozygous lines for further characterization. Consistently, both oocyst numbers and infection prevalence were significantly decreased in the miR-14 or miR-305 sponges-expressing homozygous lines when infected through a low *P*. *falciparum* gametocytaemic culture (**Fig 3C**). However, hybrids of Cp14×Vg14 or Cp305×Vg305 did not display any inhibition of the parasite in the midgut tissue (**Fig 3D**). The transgenic lines were also fed on a *P*. *berghei*-infected Swiss Webster mouse. Two miR-14 and one miR-305 sponges-expressing lines (Cp305), but not the Vg305 sponges-expressing line, showed significantly reduced *P*. *berghei* oocyst numbers (**Fig 3E**). The differential suppression of *P*. *falciparum* and *P*. *berghei* in the Vg305 sponges-expressing line can likely be attributed to differences in how the mosquito’s immune system influences midgut invasion and other biological features of the human and rodent malaria parasites [28]. Depletion of miR-14 or miR-305 also resulted in a profound decrease in *P*. *falciparum* sporozoite-stage parasites in the salivary gland (**Fig 3F**), which could be the result of a decrease in parasite oocyst numbers in the midguts of transgenic lines.

### Sponges-mediated depletion of aga-miR-14 and aga-miR305 enhances the mosquitoes’ antibacterial defenses

**Fig 4:**
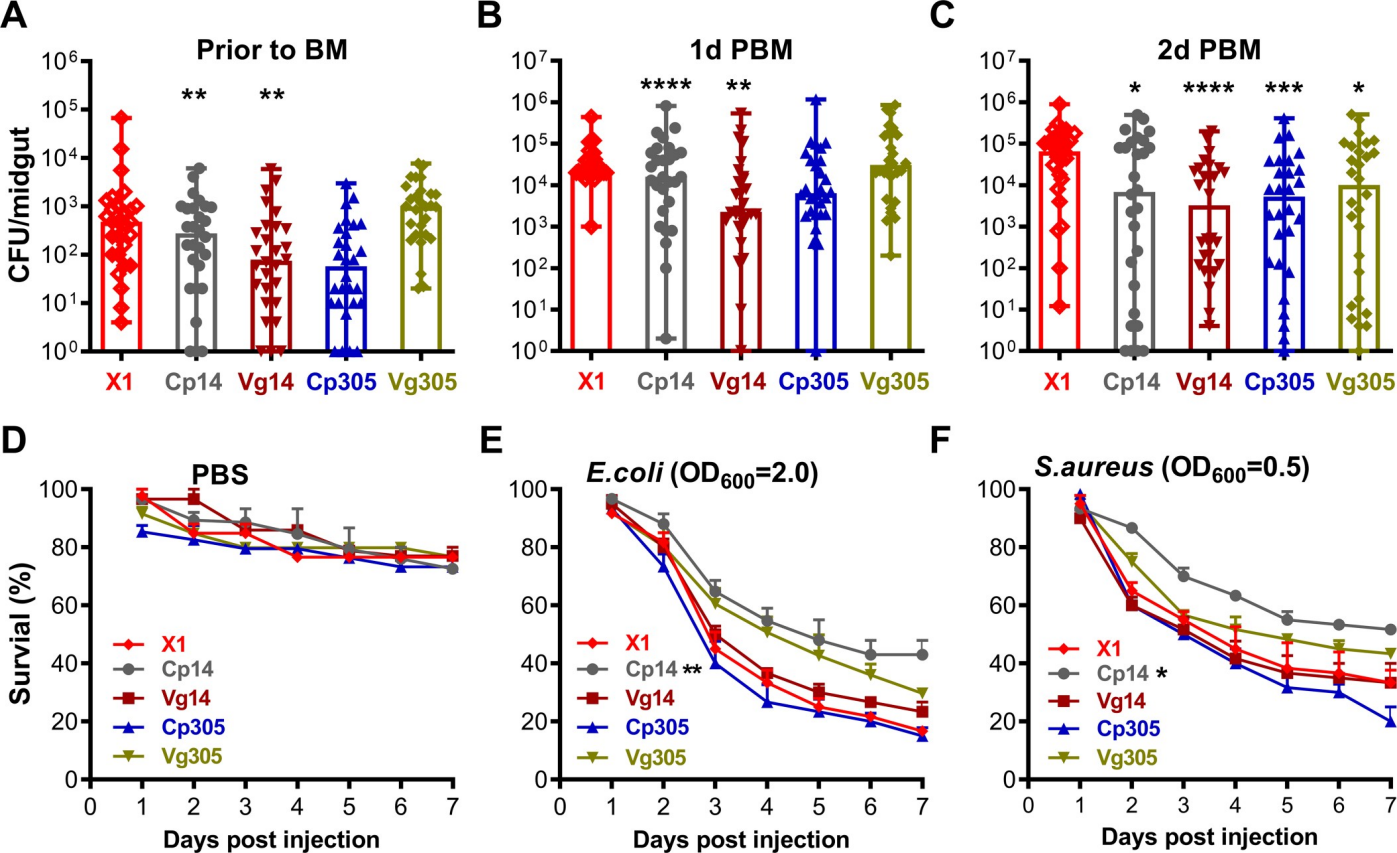
Midgut microbiota loads are significantly reduced in miRNA-SPs overexpressing transgenic *An*. *gambiae*, and mosquitoes become more resistant to systemic bacterial challenge. Points indicate the absolute value of cultivated microbiota colony-forming-units (CFUs) in individual midguts of mosquitoes before blood feeding (**A**) or at 1 day (**B**) or 2 days (**C**) PBM, and error bars in each column represent the median value with the range of microbiota CFU. Statistical comparisons of cultivated microbiota between transgenic lines and the docking line (X1) were performed using the Mann-Whitney test. *P<0.05, **P<0.01, ***P<0.001, ****P<0.0001. Survival rate of mosquitoes that were injected with PBS (**D**), *E*. *coli* (**E**), or *S*. *aureus* (**F**) at 3–6 h PBM. Statistical comparisons of mortality between transgenic and X1 lines were performed using the logrank (Mantel-Cox) test. *P<0.05, **P<0.01.

The mosquito midgut microbiota needs to be continuously controlled by the mosquito’s innate immune system to avoid over proliferation that could result in a lethal infection [7]. Since both miR-14 and miR-305 could target multiple immunity genes, we assessed the effect of sponges-mediated miR-14 and miR-305 depletion on the midgut microbiota load. The cultivable bacterial load of midguts from individual transgenic and control females was determined just prior to blood feeding (BF) and at day 1 and 2 PBM. The cultivable midgut bacteria load of the Vg14 and Cp305 lines were significantly reduced compared to those of the X1 control mosquitoes (**Fig 4A and 4B**). At 2 days PBM, all the transgenic mosquitoes showed a significantly suppressed midgut bacteria load (**Fig 4C**), suggesting that depletion of miR-14 and miR-305 resulted in an enhancement of the midgut antibacterial defense. We also investigated whether sponges-mediated depletion of miR-14 and miR-305 in either the midgut or fat body tissues would result in a greater resistance to systemic bacterial infection. Transgenic mosquitoes were systemically challenged (by injection) with either the Gram-negative bacterium *Escherichia coli* or the Gram-positive bacterium *Staphylococcus aureus*, and dead mosquitoes were recorded daily until day 7 post-injection. Compared to the X1 control, only one miR-14-SPs-expressing line (Cp14) exhibited significantly increased resistance to systemic *E*. *coli* (p<0.05) and *S*. *aureus* (p<0.05) infection (**Fig 4D–4F**). Together, our data suggests that the sponges-mediated depletion of miR-14 or miR-305 resulted in an enhanced antibacterial activity in mosquitoes.

### The mRNA abundance of immune and anti-*Plasmodium* genes is increased in midguts upon sponges-mediated depletion of aga-miR-14 and aga-miR-305

**Fig 5:**
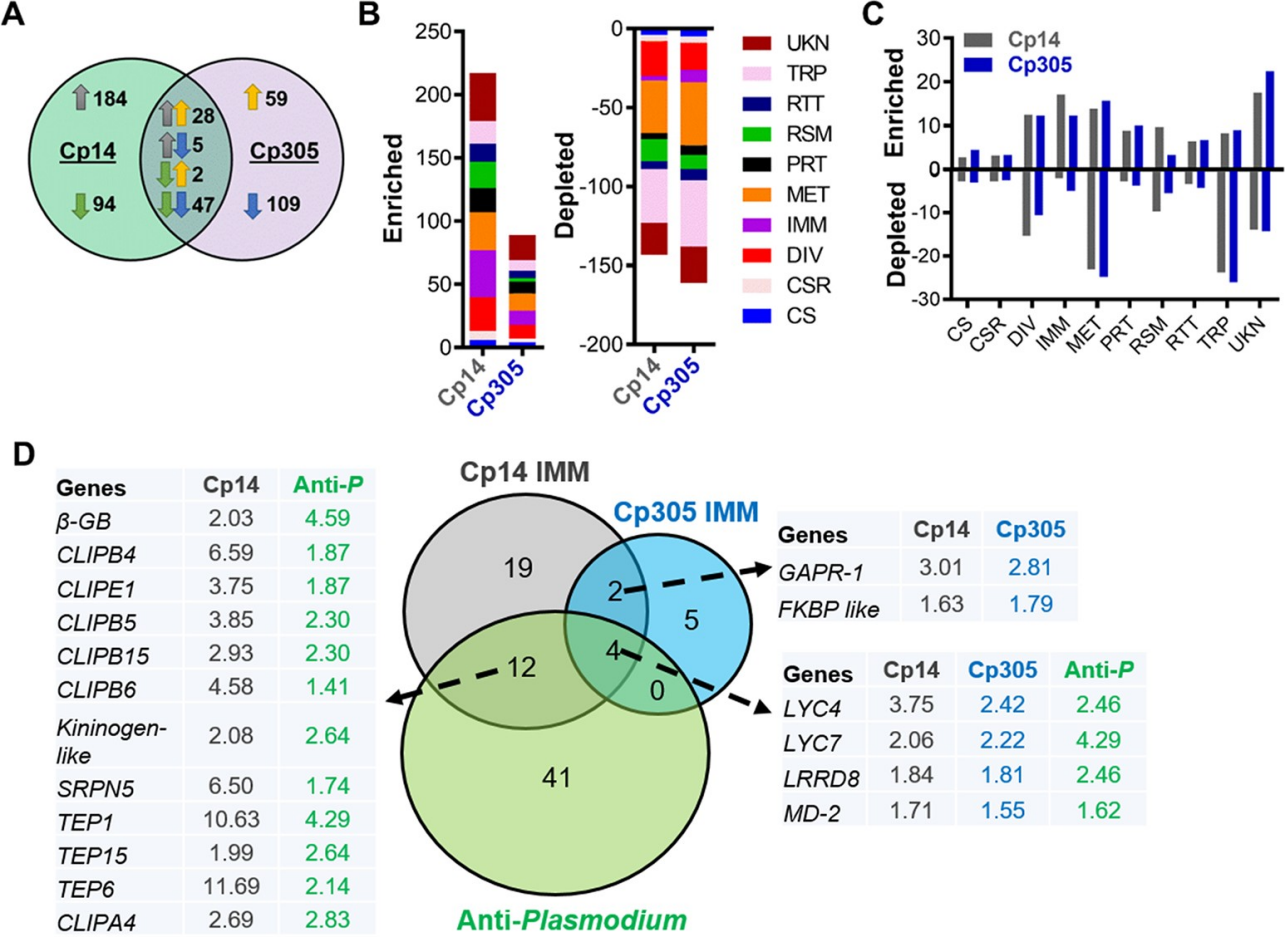
Midgut transcriptomic profiles are altered upon sponges-mediated depletion of aga-miR-14 or aga-miR-305 at 1 day PBM. (**A**) Venn diagram showing the number of shared and unique differentially expressed (DE) genes (fold change >1.5, *P*<0.05). The green circle represents the number of DE genes from the Cp14 line, and the purple circle represents those from the Cp305 line. Upward arrows represent upregulated DE genes, and downward arrows represent downregulated DE genes. (**B**) Number of DE genes in the midguts of the transgenic and X1 lines. Functional groups were classified as previously described. Abbreviations: CS, cytoskeletal and structural; CSR, chemosensory reception; DIV, diverse functions; IMM, immunity; MET, metabolism; PRT, proteolysis; RSM, redox, stress, and mitochondrion; RTT, replication, transcription, and translation; TRP, transport; UKN, unknown functions. (**C**) Percentage of upregulated or downregulated DE genes in the midguts of transgenic lines compared to the X1 line, for each functional group. (**D**) Venn diagram showing the number of shared and uniquely upregulated anti-*Plasmodium* genes in both Cp14 and Cp305 midguts. The gray circle represents the number of upregulated immunity DE genes in the Cp14 midguts, the blue circle represents the upregulated immunity DE genes in the Cp305midguts, and the green circle represents anti-*Plasmodium* transcripts identified in the midguts of *An*. *gambiae* by Dong *et al*. 2006 [28]. The tables show the fold change in upregulated shared genes. Unlike ‘CP14 IMM’ and ‘Cp305 IMM’, the fold changes for ‘anti-*Plasmodium*’ resulted from a comparison of the infected and uninfected mosquitoes. Abbreviations: *β-GB*: beta-1,3-glucan-binding; *CLIPA/B*: CLIP-domain serine proteases; *BDBT FKBP like*: FK506-binding proteins; *LRRD8*: leucine-rich repeat domain 8; *LYSC*: C-type lysozyme; *MD-2*: Niemann-Pick type C-2; *GAPR-1*: Golgi-associated plant pathogenesis-related protein 1; *Tep*: thioester-containing protein.

To identify genes whose mRNA abundance was influenced by sponges-mediated depletion of miR-14 or miR-305 in the midgut tissue, we performed an RNA-Seq-based midgut transcriptome analysis of the Cp14, Cp305, and X1 lines at 24 h PBM **(S4 Table)**. A total of 360 differentially expressed (DE) (-fold change >1.5, *P*<0.05) genes (217 upregulated and 143 downregulated) and 250 DE genes (89 upregulated and 161 downregulated) were observed in the midguts of the Cp14 and Cp305 mosquitoes, respectively **(Fig 5A and S5 Table)**. In the midguts of both transgenic mosquito lines, 28 and 47 DE genes were upregulated and downregulated, respectively, when compared to X1 control mosquitoes **(Fig 5A)**. The majority of these DE genes matched the following functional GO categories: Metabolism, Immunity, Transport, and Unknown Functions **(Fig 5B)**. An increased abundance of transcripts belonging to the functional GO categories Immunity and Proteolysis and a decreased abundance of transcripts belonging to the category Transfer were observed in the midguts of transgenic mosquitoes **(Fig 5C)**.

There were 40 (37 upregulated versus 3 downregulated) and 18 (11 upregulated versus 7 downregulated) genes in the midguts of Cp14 and Cp305 mosquitoes, respectively, belonging to the Immunity DE gene category **(Fig 5B and S5 Table)**. Among the upregulated Immunity DE genes, 6 shared genes were observed in Cp14 and Cp305. The midguts of Cp14 and Cp305 also displayed 16 and 6 Immunity DE genes, respectively, with putative anti-*Plasmodium* activity [28] **(Fig 5D)**.

### Overall fitness cost is low upon sponges-mediated depletion of aga-miR-14 or aga-miR-305

**Fig 6:**
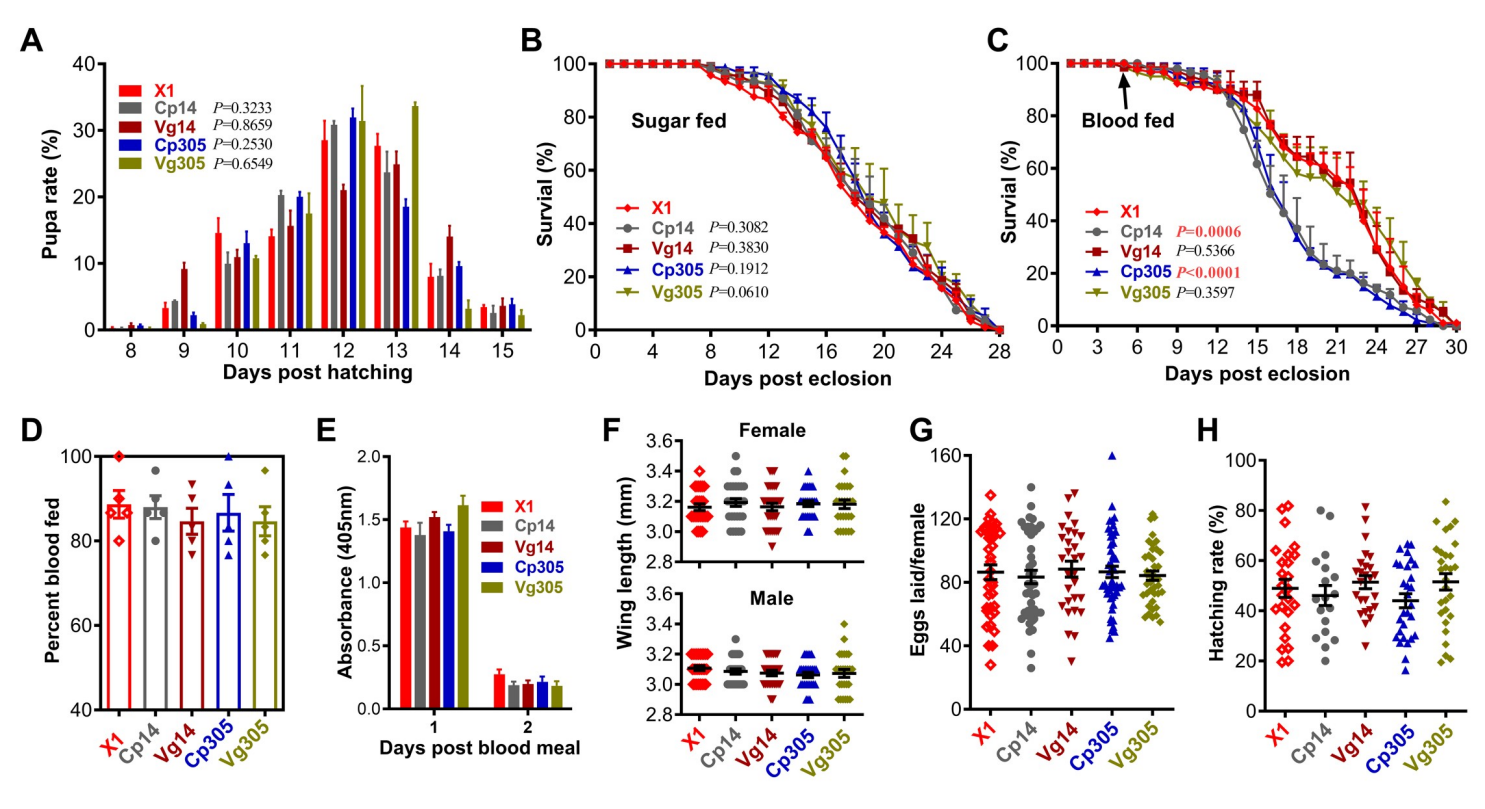
Fitness cost of miRNA-SPs overexpressing transgenic *An*. *gambiae*. (**A**) The pupation time did not differ between the transgenic lines and the docking line (X1). The *P*-value was determined by the Gehan-Breslow-Wilcoxon test. Female longevity of transgenic mosquitoes was not affected when mosquitoes were fed on a regular sugar meal (**B**), but it was significantly decreased in the Cp14 and Cp305 lines after a naïve blood meal (**C**). The *P* value was determined by the logrank (Mantel-Cox) test. Female blood-feeding ability (**D**), endogenous trypsin activity of midguts at 1 day and 2 days PBM (E), wing length of females and males (**F**), eggs laid per female (**G**), and hatching rate (**H**) were not affected by conditional depletion of miR-14 or miR-305. Points indicate the absolute value of each parameter, and horizontal black bars in each column represent the mean value with standard error (SE). Statistical comparisons between transgenic and X1 lines were performed using the Mann-Whitney test.

Multiple parameters were used to determine the impact of miRNA depletion on mosquito fitness. We first compared the larval development and pupation time between the transgenic and X1 lines and no significant differences were observed (**Fig 6A**), consistent with the properties of the *Cp* and *Vg* promoters that are predominantly induced after a blood meal in adult females. The survival rate of adult females maintained on a 10% sucrose solution or receiving a naïve blood meal at 5 days post-eclosion were also recorded to evaluate longevity. There were no significant differences in the longevity of sugar-fed transgenic females between any of the lines (**Fig 6B**); however, adult females of the Cp305 and Cp14 lines showed a significantly shortened life span when compared to X1 mosquitoes (**Fig 6C**) after having been provided a naïve blood meal at 5 days post-eclosion. The blood-feeding ability of mosquitoes on mice did not differ between the transgenic mosquitoes and X1 females (**Fig 6D**), and endogenous trypsin activity was not affected in midguts of transgenic mosquitoes at 1 day and 2 days PBM when compared to X1 control (**Fig 6E**). Wing length, an indicator of the body size of the mosquitoes, was also not affected by the sponges-mediated depletion of miR-14 or miR-305 in either female or male adults (**Fig 6F**). Female fecundity, measured as the number of eggs produced per female and their hatching rate, did not differ between the transgenic lines and X1 mosquitoes (**Fig 6G and 6H**).

## Discussion

Mosquitoes possess a highly conserved miRNA biosynthesis pathway, and the *An*. *gambiae* genome contains at least 168 miRNAs [16, 29] displaying spatiotemporally expressed specificity [11]. However, only a few miRNAs have been functionally characterized in malaria mosquitoes [14–16]. Here, we investigated the function of miRNAs in regulating *P*. *falciparum* infection and the midgut microbiota. We used a CLEAR-CLIP approach to first identify *An*. *gambiae* miRNAs that bind to mRNA targets, then specifically focused on those miRNAs targeting immunity genes and showing differential abundance between *P*. *falciparum*-infected and naïve blood-fed mosquitoes. Conditional depletion of aga-miR-14 or aga-miR-305, but not aga-miR-8, resulted in a suppression of both *P*. *falciparum* and *P*. *berghei* infections, as well as midgut microbiota. The lack of infection phenotype for the aga-miR-8 sponges-expressing line suggests that this miRNA is not implicated in the anti-*Plasmodium* defense reaction, but rather in some other physiological system that may respond to infection. The lack of infection phenotypes of the transheterozygote hybrids of Cp14×Vg14 or Cp305×Vg305 is likely due to insufficient production of miRNA sponges by the single allele. The mosquito’s innate immune system can discriminate between these two parasite species, and our earlier studies have shown that the IMD pathway is more specifically involved in suppressing *P*. *falciparum*, whereas the Toll pathway is predominantly involved in mediating defense against *P*. *berghei* infection [28, 30]. The suppression of both parasite species and the microbiota after aga-miR-14 and aga-miR-305 depletion suggests that these miRNAs regulate the activity of diverse immune genes, thereby playing a role in modulating broad-spectrum defenses. Transcriptome analysis suggested that the anti-*Plasmodium* activity mediated by the miR-SPs resulted from an increased activity of multiple anti-*Plasmodium* effector gene mRNAs. For example, the mRNA abundance of the key anti-*Plasmodium* factor *Tep1* [31] increased more than 10-fold in mosquito midguts upon depletion of aga-miR-14.

Hundreds of genes were significantly regulated in the midguts of transgenic mosquitoes upon depletion of aga-miR-14 or aga-miR-305; however, only a few of these genes contained potential binding sites for the miRNAs studied here. This is not surprising given the broad physiological effects of miRNA depletion that affected permissiveness to *Plasmodium*, survival upon bacterial infection, midgut microbiota load as well as fecundity as a measure of egg laying and hatch rates. The majority of regulated genes reflect secondary effects of miRNA targeted mRNA regulation, and our RNAseq-based transcriptome approach measured the overall effect of miRNA depletion on mosquito physiology rather than simply identifying the differentially targeted mRNAs. Other processes contributing toward the limited number of regulated mRNA with miRNA binding sites are likely to be a poor degradation of the targeted mRNAs identified by CLEAR-CLIP, mRNA replenishment by newly transcribed RNA, and it is also possible that miR-14 and miR-305 inhibit gene expression primarily via translational repression instead of mRNA degradation [32, 33]. Furthermore, it is also possible that differential expression of certain genes identified by our transcriptome analyses resulted from non-canonical seed pairing rules [34, 35]. Some of the miRNA-mRNA interactions identified by CLEAR-CLIP may also have taken place in mosquito tissues other than the midgut.

Transgenesis of any organism frequently results in fitness costs [36–38]. Both miR-14 and miR-305 are required for maintaining longevity in *Drosophila melanogaster*. MiR-14 is a cell death suppressor, and miR-14 mutants are stress-sensitive and have a reduced lifespan [39]. *Drosophila* miR-305 is required for adaptive homeostasis of the gut and functions by regulating the Notch and insulin pathways in intestinal stem cells; when miR-305 is depleted, the life span of adult flies is decreased [40]. To minimize possible fitness costs, we used promoters with spatiotemporal expression specificity to drive the production of miR-SPs. Only the longevity of the blood-fed females was affected, and increased mortality was only observed after 8 days PBM to complete at least one gonadotrophic cycle, allowing the mosquitoes to lay eggs. All other tested parameters were unimpaired in all strains of transgenic mosquitoes.

Mosquito population replacement is emerging as a promising malaria control strategy and has gained increasing attention with the development of gene-drive technologies [36, 41–44]. The successful implementation of a population replacement strategy requires the development of *Plasmodium*-resistant transgenic mosquitoes. Ideally, the transgenic mosquitoes should have multiple blocking mechanisms to make it difficult for the parasite to develop resistance and also to make blocking more effective. An advantage of our miR-SPs–based approach is the relatively small size of the construct and the regulation of multiple anti-*Plasmodium* genes. Resistance to *Plasmodium* could potentially be enhanced by targeting multiple miRNAs through combinations of different sponges. Combining miR-SPs with *Plasmodium* antagonist overexpression, or host factor deletion, is also likely to result in a potentiated parasite suppression made possible by multiple blocking-mechanisms.

## Material and methods

### Ethics statement

This study was carried out in strict accordance with the recommendations in the Guide for the Care and Use of Laboratory Animals of the National Institutes of Health. The protocol was approved by the Animal Care and Use Committee of the Johns Hopkins University (permit number MO15H144). Commercial anonymous human blood was used for parasite cultures and mosquito feeding, and informed consent was therefore not applicable. The Johns Hopkins Bloomberg School of Public Health Ethics Committee has approved this protocol.

### CLEAR-CLIP library construction

Specific polyclonal antibody against *An*. *gambiae* Ago1 was produced by Fu *et al*., 2017 [9]. The experimental protocol was adapted from previous reports [21, 45]. For each sample, 150 female mosquitoes were first ground in liquid nitrogen before ultraviolet irradiation. A detailed protocol is included in **S1 Text**.

### Identification of miRNA-mRNA chimeras

The libraries were sequenced on an Illumina sequencing platform. Data were deposited into NCBI's Gene Expression Omnibus (GEO) (SRP147972). The reads were processed to remove low-quality reads and 3’ adapter sequences by using Flexbar [46]. Reads shorter than 16 nt were discarded, and reads with identical sequences were collapsed. Three random nucleotides were used in the 5’ adapter to avoid PCR over-amplification. These random barcodes were trimmed prior to mapping. The cleaned reads containing miRNA sequences were first defined by mapping mature miRNA sequences against sample libraries using BLAST (v2.3.0) with e-value equal to 0.4 [47]. Only the best alignment was reported. The remaining sequences next to the miRNAs in the reads were extracted for mapping to the *An*. *gambiae* transcripts with BLAST. The reads mapped to rRNA, tRNA and miRNA genes were removed.

### Peak calling of CLIP clusters

The peak calling of a CLIP cluster was done as previously described [19], using the pooled reads from three biological replicates at each time point. In brief, overlapping reads were collapsed to form cluster regions. Cubic spline interpolation (Scipy, http://www.scipy.org/) was performed to determine the location and read number of peaks within a cluster [48]. Significant peaks were identified by determining read number cutoffs with *P*-values <0.01 by Poisson distribution.

### Transgene construction and germline transformation

For the sequences of all DNA primers and miR-SPs constructs used in this study, see detail in **S2 Text**. Sponges of aga-miR-8, aga-miR-14, and aga-miR-305 were predicted using miRNAsong (http://www.med.muni.cz/histology/miRNAsong/index.php) [49] by introducing mismatches at positions 9–12 to generate a mismatch “bulge”. MiR-SPs constructs were generated with 10 (miR-14 and miR-305) or 20 (miR-8) repetitive miR-SPs sequences separated by a variable four-nucleotide linker, synthesized by GenScript, and cloned into the pUC plasmid. *Vitellogenin* (*AgVg*) and *carboxypeptidase* (*AgCp*) promoters were amplified from a cDNA library of *An*. *gambiae* with specific primers [6] and cloned into the pKSB plasmid. Each miR-SPs construct and *AgVg* or *AgCp* promoter-pKSB plasmid was assembled into a pDSAT or pDSAG plasmid through Golden Gate cloning into the *BsaI* site. The final miR-SPs plasmids and a helper plasmid carrying the *Vasa2*::ΦC31-integrase gene (pENTR-R4R3-Vasa2-integrase) were prepared with a Qiagen Endofree Maxi Kit, and a mix of final miR-SP plasmid (160 ng/μL) and helper plasmid (200 ng/μL) was micro-injected into freshly laid eggs of docking line X1 according to previously established methods [27, 38]. At least 500 embryos were injected for each miR-SPs construct (**S3 Table**). The injected eggs were kept moist on wet filter paper for 2 days before hatching in distilled water. G0 pupae were screened for transient fluorescence expression, and positive G0 pupae were divided into female and male pools before being outcrossed with X1 males or females. The G1 progeny was screened for fluorescent glowing eyes at the fourth larval stage (L4), and outcrossed with X1 or incrossed, depending on the number of G1 positives surviving.

### miRNA expression analysis

miRNA expression was analyzed quantitatively by means of the miScript PCR System from Qiagen. The fat bodies were dissected on ice by removing malpighian tubules, midgut, ovaries and crop from the abdominal section, and the remaining abdomens were used for RNA isolation. Total RNA was extracted from midguts or fat bodies of miR-SPs transgenic mosquitoes and docking line X1 by Trizol (Invitrogen), and then digested with DNase I (Invitrogen). cDNAs for miRNA analysis were produced using the miScript II RT Kit (Qiagen), and qRT-PCR was performed using the miScript SYBR Green PCR kit (Qiagen) according to the manufacturer’s protocol. Forward primers for qPCR analysis were the sequence of the mature miRNA, and reverse primers were the sequence of the miScript universal primer. All miRNA expression was plotted, using the 2^-ΔCt^ method, as expression relative to that of the internal control gene (S7).

### *P*. *falciparum* and *P*. *berghei* infection assays

One week-old adult females were starved for 4–6 h before being fed with a mixture of 0.02% (for high infection) or 0.005% (for low infection) NF54 *P*. *falciparum* gametocyte cultures (provided by the Johns Hopkins Malaria Research Institute Parasitology Core facility) with 40% washed human blood and 60% human serum through artificial membrane feeding on a glass feeder at 37°C. Fully engorged females were transferred to a new cup and kept for another 8 days at 27°C. For *P*. *berghei* infection assays, adult female mosquitoes were fed on a *P*. *berghei* wildtype ANKA 2.34-infected Swiss Webster mouse (at 19°C), and fully fed mosquitoes were kept for another 13 days at 19°C.

To determine oocyst numbers, midguts were dissected in PBS, stained with 0.2% mercurochrome, and examined using a phase-contrast microscope [6, 38]. The sporozoite loads in the salivary glands were determined according to a published method [6, 50], with modifications: Salivary glands were dissected, and individual glands were placed in a PCR tube with 30 μl of PBS, followed by homogenization on ice. A sample (10 μl) of the homogenate was placed in a Neubauer counting chamber and counted after 5 min using a Leica phase-contrast microscope at 400x magnification.

Each line had at least two or three independent replicates, and equal numbers from different replicates were randomly selected, then pooled for making graphs and conducting statistical analysis using GraphPad Prism software, based on a previously established method [6, 38, 51]. The *P*-values were determined using a non-parametric Mann-Whitney test.

### Microbiota analysis and survival assay of systemic bacterial challenge

One-week-old adult females of transgenic lines and docking line X1 were collected before blood feeding and at 1 and 2 days PBM. Mosquitoes were surface-sterilized by serial washes: first with 100% ethanol, then twice with 70% ethanol, and then with sterile PBS. Midguts were dissected from individual females in sterile PBS on a sterile Petri dish and transferred to a 1.5 ml tube containing 100 μL sterile PBS on ice. Ten-fold serially diluted midgut homogenates were plated on LB agar, and colony-forming units (CFUs) were counted after incubation for 3–4 days at room temperature (27°C). CFUs of the same shape and size were randomly selected, and 16s RNA was PCR-amplified and sequenced, then blasted against the NCBI database to identify bacterial species. Each line included 8–12 individual midguts, and three biological replicates were performed. Dot-plotted graphs were generated using GraphPad Prism software, and *P*-values were determined using a non-parametric Mann-Whitney test.

For survival assays following bacterial challenge, 5-day-old miR-SPs transgenic and X1 females were fed on anesthetized mice and challenged with bacteria or PBS (negative control) at 3–6 h PBM, as described previously [6, 28]. *E*. *coli* and *S*. *aureus* were cultured in LB broth at 37°C overnight, then washed three times with PBS and suspended in PBS; 69 nl of the bacterial suspension was injected into the mosquito’s thorax with a nano-injector (Nanoject, Drummond). Injected mosquitoes were kept in a small paper cup with moisture in an insectary. Dead mosquitoes were recorded daily until 7 days post-injection. Three replicates were performed, and each replicate included 30 females per group. The significance of the survival rate difference between transgenic and X1 mosquitoes was determined using Kaplan-Meier survival analysis with logrank (Mantel-Cox test) used for significance evaluation with GraphPad Prism software.

### RNA-Seq library preparation, sequencing, and bioinformatics analysis

Total RNA was extracted from 20 midguts of miR-SPs transgenic or X1 mosquitoes at 1 day PBM per sample using TRIzol reagent (Invitrogen, Carlsbad, CA), and three independent biological replicates were included for each sample. An Illumina sequencing library was constructed for each RNA sample according to the manufacturer’s instructions and sequenced by Novogene Co., LTD (Beijing, China) on the Illumina platform with paired-end 150 bp (PE 150). Data were deposited into NCBI's Sequence Read Archive (SRA) (PRJNA552320). Raw data in FASTQ format were processed to remove reads containing adapters, reads containing ploy-N, and low-quality reads, and clean reads were aligned to the *An*. *gambiae* genome (AgamP4.41). FeatureCounts was used to quantify transcript abundance in each sample using the gene annotation AgamP4 obtained from VectorBase, and the number of fragments per kb of transcript per million mapped reads (FPKM) for each gene was calculated, based on the length of the gene and the read count mapped to the gene. Genes with a *P*-value <0.05 and fold changes >1.5 obtained by DESeq2 were considered differentially expressed (DE) between transgenic mosquitoes and X1 controls. Gene ontology analyses of DE genes were performed using DAVID Bioinformatics Resources 6.8 (https://david.ncifcrf.gov). Over-representation of a DE gene functional category was based on previous classification [28]. Venn diagrams were generated using Venny 2.1 (http://bioinfogp.cnb.csic.es/tools/venny/index.html).

### Larval development and pupation time

Three hundred larvae were reared in a tray with fish food according to a standard procedure, and the number of pupae was recorded each day to determine development time. Each line had at least three biological replicates. Statistical significance was determined by Kaplan-Meier survival analysis (pupation treated as “dead”, equal to”1”) on pooled data from three replicates by using GraphPad Prism software, and *P*-values were determined by the Gehan-Breslow-Wilcoxon test.

### Adult survival rate

Adult females were placed into cups after emergence, and a 10% sterile sucrose solution was provided with a cotton pad and changed every 2 days. To investigate the survival percentage of blood-fed mosquitoes, 5-day-old females were fed on mice, and fully engorged females were transferred to a new cup. Each cup had 20 females, and at least three replicates were performed for each line. The number of dead mosquitoes in each cup was recorded, and dead mosquitoes were removed daily. Statistical significance was determined by Kaplan-Meier survival analysis with pooled data from three replicates by using GraphPad Prism software, and *P*-values were determined by the Gehan-Breslow-Wilcoxon test.

### Percentage of blood-feeding females

Before blood feeding, one-week old females were transferred to a small cup and starved for 4–6 h. After starvation, females were fed on anesthetized mice for 30 min, and the number of fed females was recorded. Three biological replicates were included. *P*-values between transgenic lines and the X1 line were determined using a non-parametric Mann-Whitney test.

### Endogenous trypsin activity assay

Six midguts per group were dissected on ice from mosquitoes at 1 day and 2 days PBM, and then homogenized in 120 μL of cold reaction buffer (50 mM Tris-HCl, pH 8.0, with 10 mM CaCl_2_) with a pestle. Supernatants were collected after centrifugation at 20,000*g* at 4°C for 10 min. Trypsin activity assays were performed using the synthetic colorimetric substrate Nα-benzoyl-D,L-arginine-p-nitroanilide hydrochloride (BApNA) (Sigma) [52]. The reaction mixtures, each containing 10 μl of midgut extract and 1 mM BApNA in 90 μl of reaction buffer, were then incubated at room temperature for 5 min. Absorbance values were measured in a plate reader at OD_405nm_. At least three replicates were performed.

### Wing length

Adult females and males were anesthetized on ice and kept on ice for wing length measurement. Wing length was measured manually from the distal end of the alula to the tip of the wing (without the hairy fringe) through a microscope objective containing a scale bar calibrated to a 1-mm stage micrometer. Dot-plotted graphs were generated using GraphPad Prism software, and the *P*-values between transgenic lines and X1 were determined using a non-parametric Mann-Whitney test.

### Eggs laid and hatching rate

One week-old females were fed on mice, and fully engorged mosquitoes were transferred to a new cage. At 2 days PBM, individual females were placed each in a cup containing a small oviposition water cup with moist filter paper; about 50 females were evaluated for each line. The females that died before laying eggs were removed and replaced with other blood-fed females. The number of eggs laid by each female was recorded at 4 days PBM after microscopic examination. The eggs laid from each individual female were then submerged in DI water for hatching, and larvae were counted 2 days after hatching. The hatching rate was calculated by dividing the number of larvae by the number of eggs laid by each female. Dot-plotted graphs were generated using GraphPad Prism software, and the *P*-values between transgenic lines and the X1 line were determined using a non-parametric Mann-Whitney test.

## Supporting information

S1 Fig Generation of miRNA-target chimeras to identify miRNA-mRNA interactions. (**A**) The miR-989-3p-*SIX4* interaction discovered from chimeric reads and Ago1 CLIP peaks. Sequences in green are the mature miR-989-3p. The sequences in red are mapped to the *SIX4* gene. A chimeric RNA is folded *in silico* to illustrate the intermolecular stem structure. (**B**) The Ago1 CLIP peak (red) that is mapped to the coding region of the *SIX4* mRNA overlaps with the miR-989 target site derived from the miR-989-3p-*SIX4* chimeric reads. A miR-309 target site was also detected in the 3’ UTR. (**C**) Sequence conservation of the miR-309 target in the mosquito *SIX4* genes. Orthologs of *SIX4* were recovered from several mosquito genomes and were aligned with the miR-309 target sequences identified in *Ae*. *aegypti* and *An*. *gambiae*. (**D**) A miR-8 target site was detected in the 3’ UTR of *Swim* (AGAP007684). (**E**) Alignment of the miR-8 target sites in the orthologous genes of *Swim* in different mosquito species.
(TIF)

S2 Fig Enriched miRNA-binding motifs in the chimera-defined target sites. Overrepresented motifs were discovered in multiple targets of individual miRNAs using the MEME Suite. N: number of motifs found/total number of targets analyzed. E-val: e-value of the motif returned by MEME. Most motifs are complementary to the miRNA seeds (bold). 309 target sequences were identified in *Ae*. *aegypti* and *An*. *gambiae*.
(TIF)

S3 Fig Generation and characterization of aga-miR-8 sponges overexpressing transgenic *An*. *gambiae*. (**A**) Scheme of pDSA constructs to overexpress miR-8 sponges under the control of the blood-induced midgut-specific promoter (*AgCp*) or fat body-specific promoter (*AgVg*). Abbreviations: AgCp, *An*. *gambiae carboxypeptidase* promoter; AgVg, *An*. *gambiae vitellogenin* promoter; sv40, polyadenylation signal of the simian virus 40 VP1 gene; 3xP3, eye tissue-specific promoter; 20x miR-SP: 20 repetitive aga-miR-8 complementary sequences separated by variable four-nucleotide linker sequences; attB, phage Φ31 attB site. (**B**) miR-8 binding sites with a four-nucleotide central bulge. (**C**) *AgVg* control of the miR-8 sponge transgenic line with blue fluorescence marker, and *AgCp* line with the green fluorescence marker. The docking line X1 has no fluorescence in the eyes. (**D**) Expression of miR-8 was significantly decreased in midguts or fat bodies of transgenic mosquitoes following a blood meal. MicroRNA expression was detected by qPCR and normalized to rS7. Statistical comparisons of miRNA expression between transgenic and docking (X1) lines were performed using the unpaired t-test. *P<0.05, **P<0.01.
(TIF)

S4 Fig Characterization of miR sponges overexpressing transgenic *An*. *gambiae*. (TIF)

S1 Table Reads statistics of 12 CLEAR-CLIP libraries. (XLSX)

S2 Table CLEAR-CLIP chimeras. (XLSX)

S3 Table Data from generating miRNAs sponges overexpressing transgenic *An*. *gambiae*. Eggs of *An*. *gambiae* docking line (X1) females were co-injected with a mixture of miRNA sponge constructs and the Φ31 integrase helper plasmid. Surviving G0 larvae were individually screened under the fluorescence microscope, and the positive G0 larvae were backcrossed with X1; and positive offspring (G1) were confirmed under the fluorescence microscope.
(XLSX)

S4 Table Data quality control summary for transcriptome data. Q20: Percentages of bases whose correct base recognition rates were greater than 99% for total bases. Q30: percentages of bases whose correct base recognition rates were greater than 99.9% for total bases.
(XLSX)

S5 Table DE genes in the midguts of the Cp14 or Cp305 lines compared to the docking line (X1). DE genes: differentially expressed (DE) genes with fold change >1.5 and *P*<0.05.
(XLSX)

S1 Text The CLEAR-CLIP protocol and fitness tests of transgenic mosquitoes. (PDF)

S2 Text Sequences of the primers and plasmids. (PDF)
