# Supplementary figures and images for: Broad spectrum immunomodulatory effects of Anopheles gambiae microRNAs and their use for transgenic suppression of Plasmodium
Source: PLoS Pathog. 2020 Apr 24;16(4):e1008453. doi: 10.1371/journal.ppat.1008453 (PMC7202664; doi:10.1371/journal.ppat.1008453)

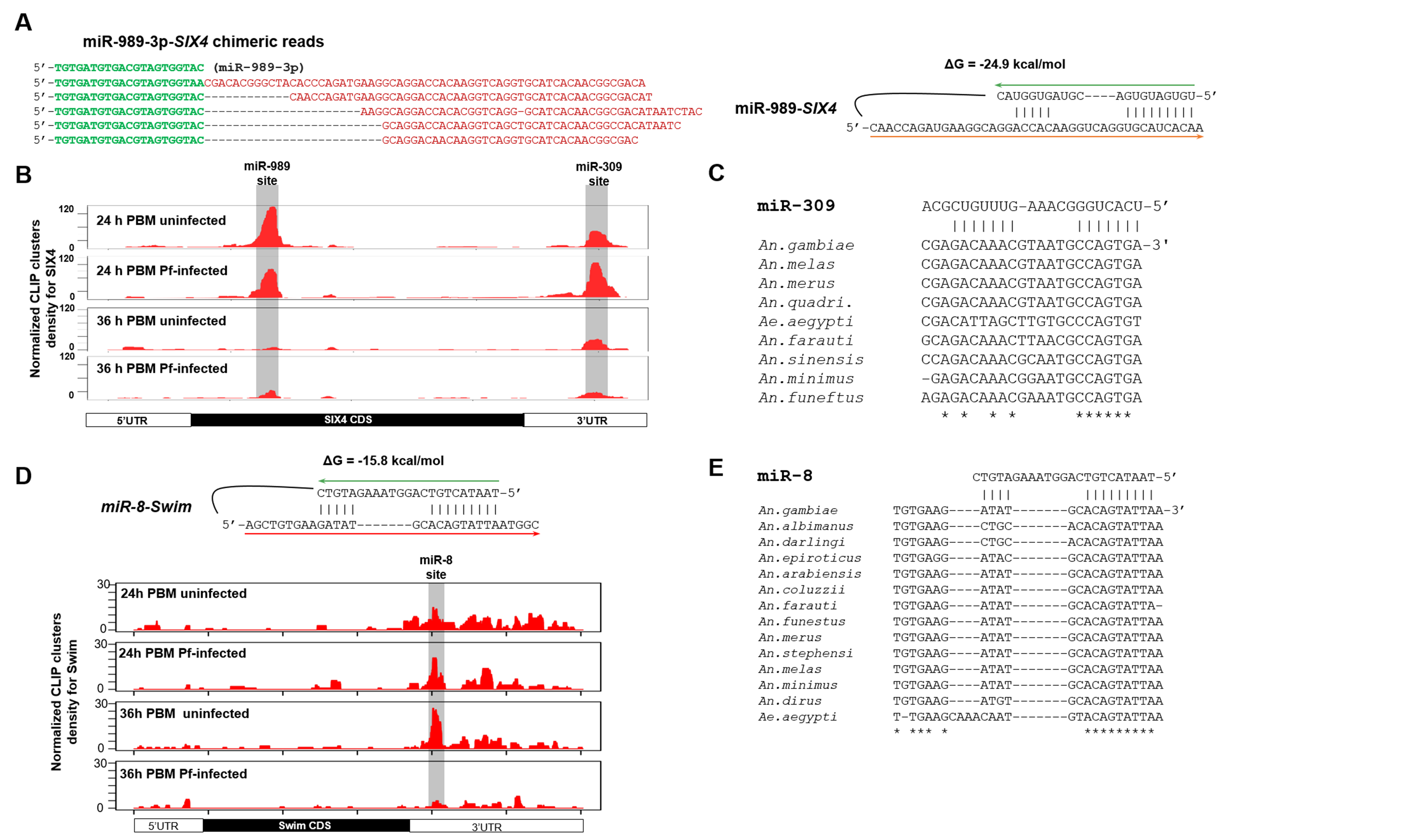

Supplement: S1 Fig — (A) The miR-989-3p-SIX4 interaction discovered from chimeric reads and Ago1 CLIP peaks. Sequences in green are the mature miR-989-3p. The sequences in red are mapped to the SIX4 gene. A chimeric RNA is folded in silico to illustrate the intermolecular stem structure. (B) The Ago1 CLIP peak (red) that is mapped to the coding region of the SIX4 mRNA overlaps with the miR-989 target site derived from the miR-989-3p-SIX4 chimeric reads. A miR-309 target site was also detected in the 3’ UTR. (C) Sequence conservation of the miR-309 target in the mosquito SIX4 genes. Orthologs of SIX4 were recovered from several mosquito genomes and were aligned with the miR-309 target sequences identified in Ae. aegypti and An. gambiae. (D) A miR-8 target site was detected in the 3’ UTR of Swim (AGAP007684). (E) Alignment of the miR-8 target sites in the orthologous genes of Swim in different mosquito species. (TIF) [file ppat.1008453.s001.tif]

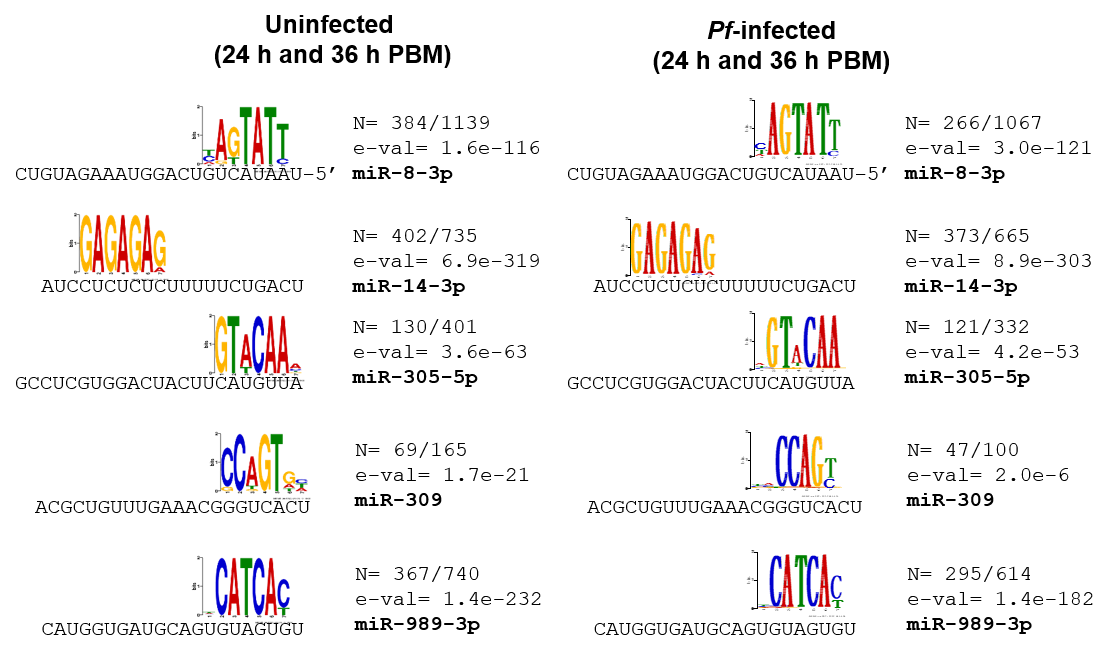

Supplement: S2 Fig — Overrepresented motifs were discovered in multiple targets of individual miRNAs using the MEME Suite. N: number of motifs found/total number of targets analyzed. E-val: e-value of the motif returned by MEME. Most motifs are complementary to the miRNA seeds (bold). 309 target sequences were identified in Ae. aegypti and An. gambiae. (TIF) [file ppat.1008453.s002.tif]

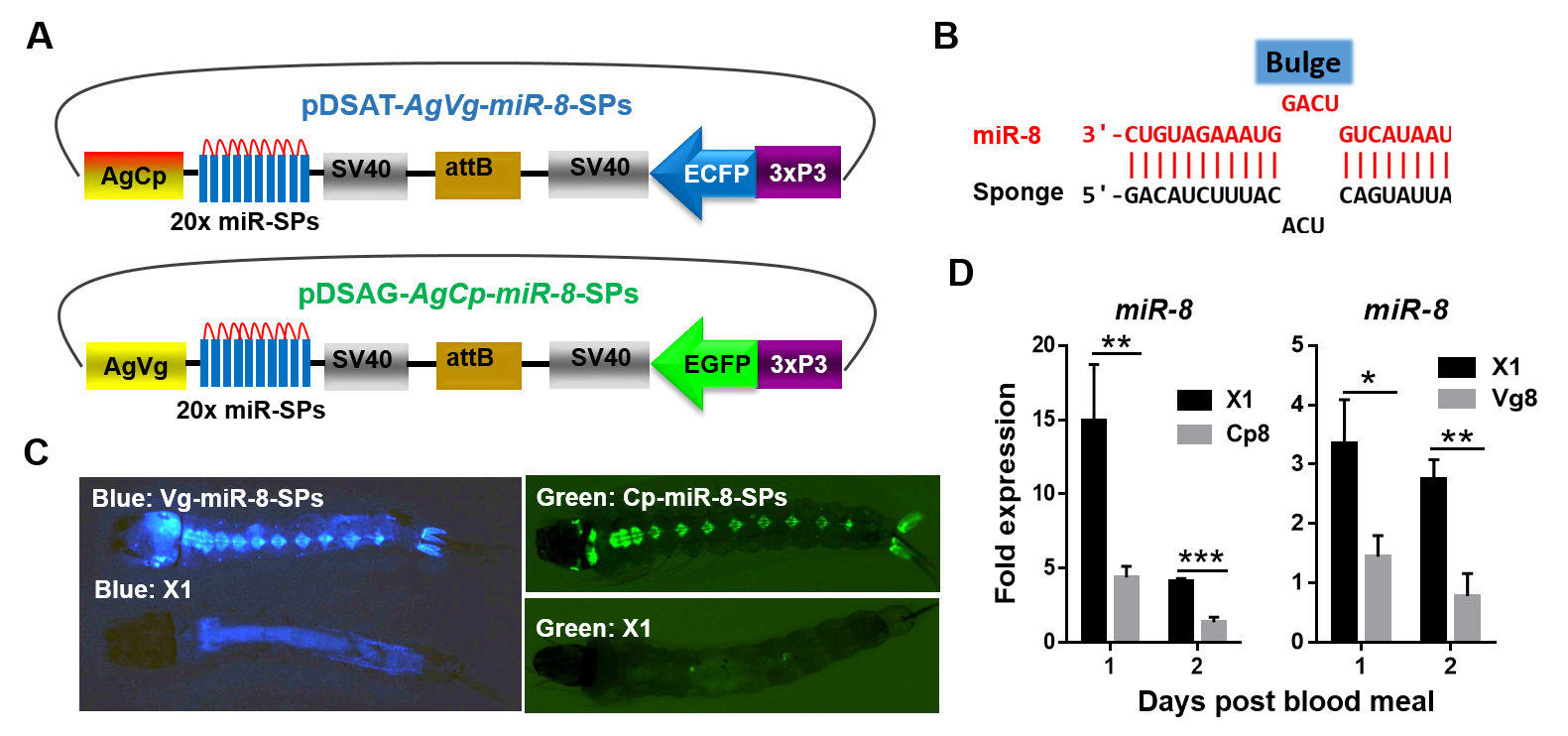

Supplement: S3 Fig — (A) Scheme of pDSA constructs to overexpress miR-8 sponges under the control of the blood-induced midgut-specific promoter (AgCp) or fat body-specific promoter (AgVg). Abbreviations: AgCp, An. gambiae carboxypeptidase promoter; AgVg, An. gambiae vitellogenin promoter; sv40, polyadenylation signal of the simian virus 40 VP1 gene; 3xP3, eye tissue-specific promoter; 20x miR-SP: 20 repetitive aga-miR-8 complementary sequences separated by variable four-nucleotide linker sequences; attB, phage Φ31 attB site. (B) miR-8 binding sites with a four-nucleotide central bulge. (C) AgVg control of the miR-8 sponge transgenic line with blue fluorescence marker, and AgCp line with the green fluorescence marker. The docking line X1 has no fluorescence in the eyes. (D) Expression of miR-8 was significantly decreased in midguts or fat bodies of transgenic mosquitoes following a blood meal. MicroRNA expression was detected by qPCR and normalized to rS7. Statistical comparisons of miRNA expression between transgenic and docking (X1) lines were performed using the unpaired t-test. *P<0.05, **P<0.01. (TIF) [file ppat.1008453.s003.tif]

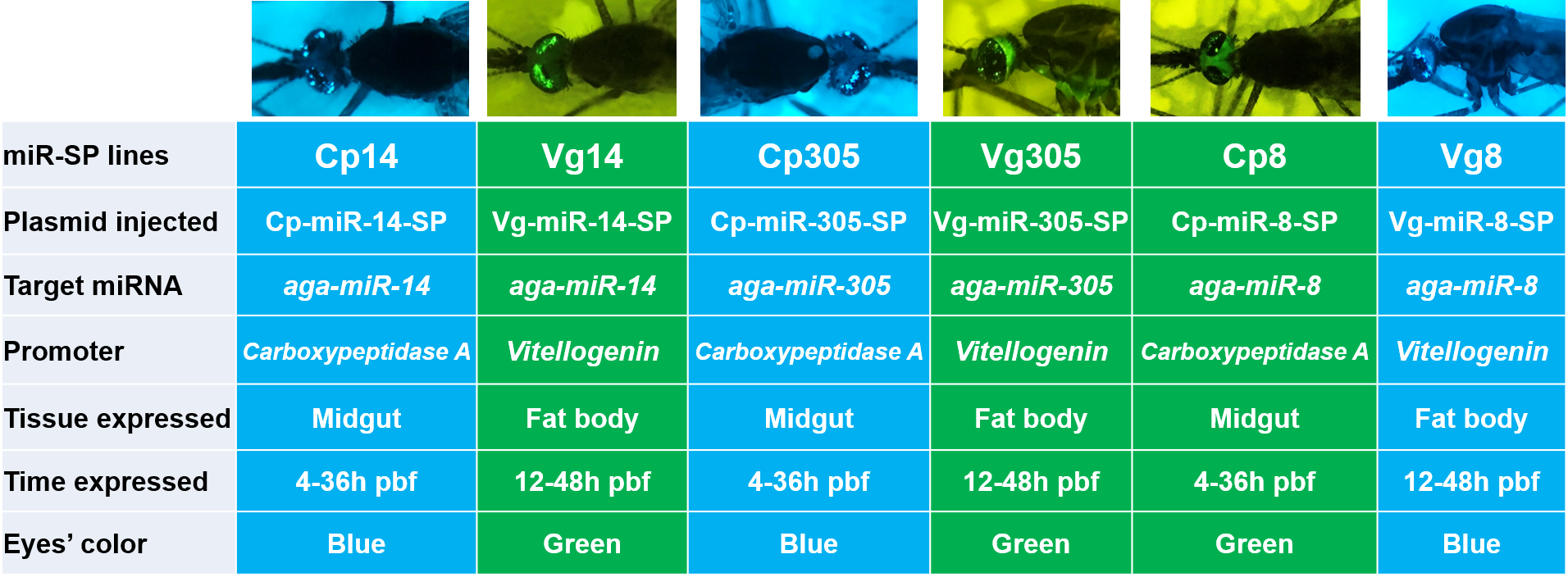

Supplement: S4 Fig — (TIF) [file ppat.1008453.s004.tif]
