# Supplementary material for: Broad spectrum immunomodulatory effects of Anopheles gambiae microRNAs and their use for transgenic suppression of Plasmodium
Source: PLoS Pathog. 2020 Apr 24;16(4):e1008453. doi: 10.1371/journal.ppat.1008453 (PMC7202664; doi:10.1371/journal.ppat.1008453)
Supplement: S1 Text — (PDF) [file ppat.1008453.s010.pdf]

Supplementary File 1: The CLEAR-CLIP protocol for *An. gambaie* Mosquitoes

Day 1

Part 1: Preparation of buffers and adapters

1) Buffers

Lysis buffer (4 °C, up to 1 month)

HBSS (pH 7.9)	50 mM
NaCl	137 mM
NP-40	1%
EDTA	5 mM
DeoxyNa	0.5%
Glycerol	10%
PMSF	5 mM
PI	2.5×
RI	0.1 U/µl

PI: Protease inhibitor (Promega)
RI: RNasin (Promega, 40 U/µl)

Tris-PXL buffer (Washing, 4 °C, up to 1 month)

Tris (pH 7.5)	25 mM
NaCl	300 mM
NP-40	0.5%
SDS	0.05%
DeoxyNa	0.5%
Glycerol	2.5%

Tris-HXL buffer (Washing, 4 °C, up to 1 month)

Tris (pH 7.5)	25 mM
NaCl	800 mM
NP-40	1.0%
SDS	0.05%
DeoxyNa	0.5%
Glycerol	2.5%

Tris-PNK buffer (Washing, 4 °C, up to 1 month)

Tris (pH 7.5)	20 mM
$MgCl_2$	10 mM
NP-40	0.5%
NaCl	50 mM

Tris-PEGTA buffer (Deactivating, 4 °C, up to 1 month)

Tris (pH 7.5)	20 mM
EGTA	20 mM

NP-40	0.5%

5× PNK buffer (Reaction buffer, -20 °C, up to 3 month)

Tris (pH 7.5)	250 mM
$MgCl_2$	50 mM
NP-40	2.5%
NaCl	250 mM
2-Mercaptoethanol	50 mM

5× PK buffer (Protease K digestion, 4°C, up to 1 month)

Tris (pH 7.5)	500 mM
NaCl	250 mM
EDTA	50 mM

2) Adaptors and primers (PAGE purified or HPLC purified):

3’ adaptor: AGATCGGAAGAGCACACGTCT-3’
5’ adaptor: GTTCAGAGUUCUACAGUCCGACGAUCNNNUAAGC-3’
SR primer: AATGATACGGCGACCACCGAGATCTACACGTTCAGAGTTCTACAGTCCGA
Index primer:
CAAGCAGAAGACGGCATACGAGATCGTGATGTGACTGGAGTTCAGACGTGTGCTCT
TCCGATC

Gel purification of adaptors

2x loading buffer (Room temperature, up to 6 month)

Formatide	95%
EDTA	10 mM

RNA elution buffer (Room temperature, up to 6 month)

Ammonium acetate	0.5 M
Magnesium acetate	10 mM
EDTA	1 mM

(1) Resuspend the adaptor to 500uM in nuclease-free water, prepare with 2x loading buffer.

(2) 10% TB-urea PAGE gel with 200 V until blue dye reach the bottom.

(3) Excise only the full-length adaptor bands (appear dark with fluorescent background).

(4) Crush the gel slurry, add 350 ul RNA elution buffer and incubate the mixture at 37 °C for 2 h or 4 °C overnight with shaking or rotating.

(5) Centrifuge to get the elution buffer with filter and precipitate by adding 1 ml of 100% EtOH.

(6) Store -20 °C 2 hr to overnight and recover the adaptors as RNA extraction procedures.

3) Preparation of ^{32}P-APP-adaptors

RL3(-P)	150 pmol
10× T4 PNK buffer	5 µl
^{32}P-γ-ATP	20 µl
T4 PNK (NEB)	6 µl
d_2H_2O	12 µl
-------------------------------	
	50 µl

Incubate at 37 °C for 30 min. add 2 µl 1 mM ATP @37 °C for 5 min. Purify using ZYMO Oligo clean and concentrator, elute with 10 µl d_2H_2O.

^{32}P-RL3	150 pmol
10× 5'APP buffer	3 µl
ATP 1 mM	3 µl
Mth ligase (NEB)	3 µl
d_2H_2O	11 µl
--	
	30 µl

Incubate at 65 °C for 60 min. Purify using ZYMO Oligo clean and concentrator, elute with 20 µl d_2H_2O and store in -80 °C.
[The ^{32}P-APP-adaptors should be carefully stored following the rule of radioactive material; they should be used within one week]

Day 2
Part 2: UV cross-linking

(1) Collect sufficient mosquitoes (~100 of 24h PBM, ~150 NBF), use a mortar and pestle to grind the sample three times with liquid N_2. The mortar and pestle were pretreated with RnaseZap and chilled with liquid nitrogen.

(2) Pre-chill a petri dish in liquid nitrogen and transfer the ground sample into the petri dish while in liquid nitrogen.

(3) Irradiate the suspension three times in a SPECTROLINKER XL-1000 (254 nm) at the energy level 400 mJ/cm^2 with 1-minute break. Mix the suspension using pipette between the irritations.

SPECTROLINKER XL-1000 setting:
<1>, Warm-up 5 minutes through **Optimal Crosslink** mode, from a cold start before using the unit.
<2>, Put the sample in the middle of the container, and press **Energy Mode,** input the number of energy level, press **START**.

<3>, After the dosage delivering, open the door and mix the suspension and resume the operation.

Part 3: Cell lysis, partial RNA digestion and immunoprecipitation

(1) Add 1 ml Cell Lysis buffer (with protease cocktail inhibitor, 5 mM PMSF and 2.5 µl RNAsin per 1 ml extraction) to each cross-linked pellet. Resuspend it by triturating. Incubate on ice for 10 min and rotate for 10 min at 4 °C.

(2) Centrifuge at 16,000 g for 30 min at 4 °C, transfer the supernatant to a new 1.5 ml tube.

(3) Adjust the total proteins of each sample to the same concentration. Add 30 µl of RQ1 DNase (Promega) and 2 U RNase I (**optimized concentration required**) to each tube, incubate at 37 °C for 3 min.

(4) Pre-incubate 8 µg of anti-AGO1 antibody with 50 µl Dynabeads in Cell Lysis buffer (600 µl) at 4 °C for at least 1 h. Place the tube on a magnet for 2 min to separate the beads from the solution, and discard the supernatant and resuspend the Dynabeads/antibody in 100 µl of Cell Lysis buffer.

(5) Transfer the Dynabeads/antibody suspension to the cell lysates obtained at step (5) and incubate for 1 h at 4 °C with gentle shaking.

(6) Wash the Dynabeads and associated RNA-protein complexes twice with high-salt buffer (600 µl), twice with washing buffer (600 µl), and twice with 1× PNK buffer (600 µl). Separate on the magnet between each wash, remove supernatant and resuspend by gentle pipetting.

Part 4: RNA phosphorylation

d_2H_2O	49 µl
5× PNK buffer	16 µl
ATP 10 mM	8 µl
RNasin	2 µl
T4 PNK	4 µl
--------------------------------	
	80 µl

(1) Incubate in Thermomixer R at 20 °C for 150 min.
(2) Wash twice with PXL Wash Buffer.
(3) Wash twice with PNK Buffer.

Part 5: Intermolecular ligation

d_2H_2O	104 µl
5×PNK buffer	32 µl

ATP 10mM	16 µl
RNasin	4 µl
T4 RNA ligase1	4 µl

----

160 µl

(1) Incubate in Thermomixer R at 16 °C for at least 12 h (overnight).
(2) Wash twice with PXL Wash Buffer.
(3) Wash twice with PNK Buffer.

Day 3

Part 6: RNA dephosphorylation

d_2H_2O	54 µl
5× PNK buffer	16 µl
RNasin	2 µl
TSAP (Promega)	8 µl

----

80 µl

(1) Incubate in Thermomixer R at 20 °C for 45 minutes.
(2) Wash twice with PXL Wash Buffer.
(3) Wash twice with PNK Buffer.

Part 7: 3'-RNA linker ligation(On-Beads)

d_2H_2O	32 µl	
5× PNK buffer	16 µl	
PEG 400	20 µl	
RNasin	2 µl	
^{32}P-APP-linker		20+60 pmol
T4 RNA ligase2 (K227Q)	4 µl	

----

80 µl

Add 80 µl of ligase mix to each tube of beads.

Incubate at 16 °C for 1 h in Thermomixer R (1000 rpm every 2 minutes for 15 seconds). Add 60 pmol of RL3, and let the reaction go for 5 h.

Part 8: SDS-PAGE & nitrocellulose transfer

(1) Re-suspend the beads in 30 µl of Novex loading buffer.

(2) Load 30 µl of sample per well on Novex NuPAGE 4-12% Bis-Tris gel. Run the gel at 175 V for 3 h.
(3) After the gel run, transfer the gel to S&S BA-85 nitrocellulose using the Novex wet transfer apparatus.
(4) Transfer at 30 V for 2 h in NuPAGE Transfer Buffer with 10% methanol.
(5) After the transfer, rinse the nitrocellulose filter in 1x PBS, and gently blot on Kimwipes; wrap membrane in plastic wrap and expose to autoradiogram.

[The exposing time depends on the intensity of the signal. Strong band within 24 h exposing should provide sufficient RNAs for library preparation.]

Day 4

Part 9: RNA isolation and purification

(1) Make a 4 mg/ml proteinase K solution in 1× PK buffer; pre-incubate this stock in 37 °C for 20 min to eliminate l any RNases.
(2) Add 200 µl of Proteinase K solution to each tube of isolated nitrocellulose pieces; incubate for 20 min at 37 °C at 1000 rpm.
(3) Add 200 µl 1× PK/7 M urea solution; incubate for another 20 min at 37 °C at 1000 rpm.
(4) Add 600 µl of RNA phenol:$CHCl_3$ (5:1) to solution; 37 °C for 20 min at 1000 rpm.
(5) Spin tubes at full speed in centrifuge; transfer aqueous phase to a new tube.
(6) Add 50 µl (1/10 volume) of 3 M NaOAc (pH 5.2), 0.75 µl of glycogen and 1 ml of 1:1 EtOH:isopropanol, mix thoroughly.
(7) Precipitate overnight at -20 °C.
(8) Spin down RNA for 30 min at max speed (16000 g) in a microcentrifuge. Wash pellet once with 1 ml of 75% chilled ethanol (-20 °C), and quick spin to remove the remaining ethanol. Air-dry for 5-10 min at room temperature.

Part 10: 5' RNA linker Ligation

Re-suspend the RNA in 11 µl H_2O. Add the following mixture

PNK treatment:

10× ligation buffer	1.5 µl
ATP 10 mM	1.5 µl
RNasin	0.5 µl
T4 PNK	1.0 µl
--	
	15 µl

Incubate at 37 °C for 30 minutes.

Add the ligation mixture

5’ ligation:

10× ligation buffer	0.5 µl
ATP 10 mM	0.5 µl

T4 RNA ligase 1	1.0 µl
5'-adaptor	1.0 µl (10 pmol)
d_2H_2O	2.0 µl
--	
	20 µl

Incubate at 16 °C for more than 6 h. Add 380 ul nuclease-free water to re-purify the RNA. Precipitate the RNAs in -20 °C for at least 2 h.

Day 5

Part 11: RT-PCR and PCR amplification to incorporate index for the final library

(1) Re-suspend the purified RNAs in 8 µl water.
Reverse transcription:

RNA	8.0 µl
dNTP 10 mM	1.0 µl
SR RT primer	1.0 µl (10 µM)
--	
	10 ul

Incubate at 80 °C for 3 min, put on ice immediately for more than 1 min. Add the following mixture.

10× RT buffer	2 µl
DTT	2 µl
RNasin	1 µl
$MgCl_2$	4 µl
--	
total	19 µl

Incubate at 50 °C for 3 min, then add 1 µl RT enzyme, incubate at 50 °C for 1 h.

(2) PCR to add index:

Template	10 µl
pfx master mix	185 µl
SR primer	2.5 µl (10 µM)
index primer	2.5 µl (10 µM)
--	
	200 µl

94 °C 2 min
98 °C 20 s, 58 °C 20 s , 68 °C 20 s x 19-24 cycles
68 °C 5 min
