# Supplementary material for: Broad spectrum immunomodulatory effects of Anopheles gambiae microRNAs and their use for transgenic suppression of Plasmodium
Source: PLoS Pathog. 2020 Apr 24;16(4):e1008453. doi: 10.1371/journal.ppat.1008453 (PMC7202664; doi:10.1371/journal.ppat.1008453)
Supplement: S2 Text — (PDF) [file ppat.1008453.s011.pdf]

```
LOCUS       Exported                6788 bp ds-DNA    circular SYN 24-MAY-2019
DEFINITION  .
ACCESSION   .
VERSION     .
KEYWORDS    pDSAT-AgCp-aga-miR-14 sponges
SOURCE      synthetic DNA construct
  ORGANISM  synthetic DNA construct
REFERENCE   1  (bases 1 to 6788)
  AUTHORS   .
  TITLE     Direct Submission
  JOURNAL   Exported Friday, May 24, 2019 from SnapGene 2.3.2
            http://www.snapgene.com
FEATURES             Location/Qualifiers
     source          1..6788
                     /organism="synthetic DNA construct"
                     /mol_type="other DNA"
     misc_feature    271..286
                     /note="M13F"
     misc_feature    472..2794
                     /note="AgCp promoter"
     misc_feature    2795..3030
                     /note="aga-miR-14 sponges"
     misc_feature    3036..3262
                     /note="SV40 term"
     misc_feature    3263..3539
                     /note="attB"
     misc_feature    3613..3662
                     /note="3x Pax6 binding sites"
     misc_feature    3702..3709
                     /note="TATA"
     misc_feature    3873..4589
                     /note="mTurquoise2"
     misc_feature    4618..4839
                     /note="SV40 term"
     promoter        complement(5030..5048)
                     /note="T7 promoter"
                     /note="promoter for bacteriophage T7 RNA polymerase"
     misc_feature    complement(5049..5067)
                     /note="M13R"
     misc_feature    complement(5093..5111)
                     /note="DONR-RP"
     CDS             5182..5991
                     /codon_start=1
                     /gene="aph(3')-Ia"
                     /product="aminoglycoside phosphotransferase"
                     /note="KanR"
                     /note="confers resistance to kanamycin in bacteria or G418
                     (Geneticin(R)) in eukaryotes"
                     /translation="MSHIQRETSRPRLNSNMDADLYGYKWARDNVGQSGATIYRLYGKP
                     DAPELFLKHGKGSVANDVTDEMVRLNWLTEFMPLPTIKHFIRTPDDAWLLTTAIPGKTA
```

```
                     FQVLEEYPDSGENIVDALAVFLRRLHSIPVCNCPFNSDRVFRLAQAQSRMNNGLVDASD
                     FDDERNGWPVEQVWKEMHKLLPFSPDSVVTHGDFSLDNLIFDEGKLIGCIDVGRVGIAD
                     RYQDLAILWNCLGEFSPSLQKRLFQKYGIDNPDMNKLQFHLMLDEFF"
ORIGIN
        1 ctttcctgcg ttatcccctg attctgtgga taaccgtatt accgcctttg agtgagctga
       61 taccgctcgc cgcagccgaa cgaccgagcg cagcgagtca gtgagcgagg aagcggaaga
      121 gcgcccaata cgcaaaccgc ctctccccgc gcgttggccg attcattaat gcagctggca
      181 cgacaggttt cccgactgga aagcgggcag tgagcgcaac gcaattaata cgcgtaccgc
      241 tagcatggat gttttcccag tcacgacgtt gtaaaacgac ggccagtctt aagctcgggc
      301 ccctacaggt cactaatacc atctaagtag ttgattcata gtgactggat atgttgtgtt
      361 ttacagtatt atgtagtctg tttttatgc aaaatctaat ttaatatatt gatatttata
      421 tcattttacg tttctcgttc aacttttcta tacaaagttg gtaccggatc caggaggcat
      481 gtcataagcc actgatgcgc ttgacaacct atgactgatt aggtctatac gcggctagaa
      541 ccgacgttgc tttgatatac gtgttgatga aaaatgcgaa tagaacctgt caagactcgg
      601 atctgatcaa tcgctcttgc tcattcctat attatttctt cttcttcttg tggctcaaca
      661 accgttgccg gtcaaggcct gtaccctttc aatgactttt ggattacccc tcatagcagg
      721 atagttggtc ctatgtatgg cggcacggtc tatttggagc ttgaacccat gacgggctta
      781 ttgttaagtc gtacgagttg acatctgtat cataagacca gctatttatc ctatattatt
      841 tacaacatgt ttaagttatg gacctgtgct ttcaatgatg tttttttttt tatttattat
      901 ttcaactatt tcctaataca ttcgtgacac gtatgtaatg atccaaacaa tatcttggct
      961 aaagcagatc gtgtcctgtt gctttgaggg acctcaataa cctacaacat tacgtgtaca
     1021 tgcggtcccc gagatacacg gtacctctta tacgcggatt cggagatacg cggttttcta
     1081 aatttgacaa ttctttgagc aaattgtact gatttgacac atcaatttca aattgccaaa
     1141 taatttccgt tttgatcgaa tgttaaaaac tatttcaaaa ggtttaaaac agttattttc
     1201 agtcagaatc atatcaaata attcataaag tgactaaaac cgccccctac ttgcaaaatt
     1261 acacaaaaat ttgtgatatt ttagcaggaa atcacgagat tcgacttacg cggaaattcg
     1321 agatacgcgg tattttgcgg ccgttttcgg tccccattaa ccgtgtattt cgggaaccgc
     1381 ctgtatatgc aagattttga gatttattca ccagctaatt ggggtggtta actttttcta
     1441 caataagaga tatgatctaa ttcttttctt ataagagtcg tcgtatctac caatagctca
     1501 caggtccttc caaatggaac cattaatatt atgtcaaaat cgttcaaaat tcaatgaaaa
     1561 cgtgtaacga ttgaatatgt gaacagcatc gatatcccct taatgcttct cgttattctt
     1621 tcaaccattc tttacatcct attggataag ttatgataga ttctcgtatt gtgtcgaata
     1681 ttgatttctt cttatattta cgatatgaga ttgtttgagg acgatttaga atgagccgat
     1741 ttgttgttaa atacatgact gaggaccaaa ttccttatct attactgcca atccccaacc
     1801 aaacgagaca ctctcatgta gcactttgtt gatcgggttt gatgcaaagc ttgaagcaag
     1861 ttcttgatag aaattaatac aaacgcaaat aacttcaact ataagcactg tatcatcgca
     1921 ctctgtcatc ttcggtaaag ccaataaacc gcctgctaaa aatatttacc cagacaatcg
     1981 tacggaattc ttcgccctca agtatcacga ttaccacgga gagtgggtgc aagcagttct
     2041 attttatgca ttcccccact cgataggaaa agcccctaca gccgctctta catgctatat
     2101 gaagtggagt ggaggcacac tttacgccgc ccgatacgca gctcggaagc agaactgttc
     2161 tgtgccggtt tggcacaaac gatgaatccg ttatgcgtac tggcaattgc gatcgcggta
     2221 ggttaatgaa cgagcaataa acattgaatt tatctctctc tccctcgatc cggggcggtg
     2281 tgtaattagc attgcacttg cccatgctct tcctaccatg atgtctaagc ccttcgtcgc
     2341 ctgcatctaa aatagatgcg cgttctgtac acgtgccttc ccgcgcatct cttgagtgca
     2401 cttgagacct tcggggtccg gtcgattatc gattatcgga aattctccac aaagcgctca
     2461 agaagtcgtg cactgggtta agctgggctc gttgtgagtt tcttcacgtt cttcccaatc
     2521 ctcttcaagc gacacattac ttatcgatta tcatcaactc agctcagccc gcaaggccgt
     2581 taaaccttgt gcgcgcgcgc gttttggccc gcatcgccaa aaggggtata aaaagcccac
     2641 tccagcagga accacccgat acagttcgtg caagcaagcg gtcgattgaa catggtgcga
     2701 ttaaacagtg cagtcggctc ccggtggtgg gccccagcga tggccatcct ggcggtggcg
```

2761 ctcagtgtcg aagcggccga ggcggatcca ctcataggag agctcaaaga ctgacgcgta
2821 ggagagctca aagactgagc cataggagag ctcaaagact gagcgatagg agagctcaaa
2881 gactgagcgt taggagagct caaagactga gcaataggag agctcaaaga ctgagcttta
2941 ggagagctca aagactgagg cataggagag ctcaaagact gaggcttagg agagctcaaa
3001 gactgagcca taggagagct caaagactga gcttctagac ataatcagcc ataccacatt
3061 tgtagaggtt ttacttgctt taaaaaacct cccacacctc cccctgaacc tgaaacataa
3121 aatgaatgca attgttgttg ttaacttgtt tattgcagct tataatggtt acaaataaag
3181 caatagcatc acaaatttca caaataaagc atttttttc actgcattct agttgtggtt
3241 tgtccaaact catcaatgta tctcgacgat gtaggtcaca gtctcgaagc cgcggtgcgg
3301 gtgccagggc gtgcccttgg gctccccggg cgcgtactcc acctcaccca tctggtccat
3361 catgatgaac gggtcgaggt ggcggtagtt gatcccggcg aacgcgcggc gcaccgggaa
3421 gccctcgccc tcgaaaccgc tgggcgcggt ggtcacggtg agcacgggac gtgcgacggc
3481 gtcggcgggt gcggatacgc ggggcagcgt cagcgggttc tcgacggtca cggcgggcaa
3541 ttcctgcaga cttccggtat ctcgcgtttg tttgatcgca cggttcccac aatggttaat
3601 tcgagctcgc ccggggatct aattcaatta gagactaatt caattagagc taattcaatt
3661 aggatccaag cttatcgatt tcgaaccctc gaccgccgga gtataaatag aggcgcttcg
3721 tctacggagc gacaattcaa ttcaaacaag caaagtgaac acgtcgctaa gcgaaagcta
3781 agcaaataaa caagcgcagc tgaacaagct aaacaatcgg ggtaccgcta gagtcgacgg
3841 taccgcgggc ccgggatcca ccggtcgcca ccatggtgag caagggcgag gagctgttca
3901 ccggggtggt gcccatcctg gtcgagctgg acggcgacgt aaacggccac aagttcagcg
3961 tgtccggcga gggcgagggc gatgccacct acggcaagct gaccctgaag ttcatctgca
4021 ccaccggcaa gctgcccgtg ccctggccca ccctcgtgac caccctgtcc tggggcgtgc
4081 agtgcttcgc ccgctacccc gaccacatga agcagcacga cttcttcaag tccgccatgc
4141 ccgaaggcta cgtccaggag cgcaccatct tcttcaagga cgacggcaac tacaagaccc
4201 gcgccgaggt gaagttcgag ggcgacaccc tggtgaaccg catcgagctg aagggcatcg
4261 acttcaagga ggacggcaac atcctggggc acaagctgga gtacaactac tttagcgaca
4321 acgtctatat caccgccgac aagcagaaga acggcatcaa ggccaacttc aagatccgcc
4381 acaacatcga ggacggcggc gtgcagctcg ccgaccacta ccagcagaac acccccatcg
4441 gcgacggccc cgtgctgctg cccgacaacc actacctgag cacccagtcc aagctgagca
4501 aagaccccaa cgagaagcgc gatcacatgg tcctgctgga gttcgtgacc gccgccggga
4561 tcactctcgg catggacgag ctgtacaagt ccggatgata gatctgacgg gtgatcaaat
4621 cagccatacc acatttgtag aggttttact tgctttaaaa aacctcccac acctccccct
4681 gaacctgaaa cataaaatga atgcaattgt tgttgttaac ttgtttattg cagcttataa
4741 tggttacaaa taaagcaata gcatcacaaa tttcacaaat aaagcatttt tttcactgca
4801 ttctagttgt ggtttgtcca aactcatcaa tgtatcttaa agcttatcga tacgcgtacg
4861 gcgcgcctag agcggccgcc accgcggtgg agctcgagta cccagctttc ttgtacaaag
4921 ttggcattat aagaaagcat tgcttatcaa tttgttgcaa cgaacaggtc actatcagtc
4981 aaaataaaat cattatttgc catccagctg cagggcggcc gcgatatccc ctatagtgag
5041 tcgtattaca tggtcatagc tgtttcctgg cagctctggc ccgtgtctca aaatctctga
5101 tgttacattg cacaagataa aaatatatca tcatgaacaa taaaactgtc tgcttacata
5161 aacagtaata caaggggtgt tatgagccat attcaacggg aaacgtcgag gccgcgatta
5221 aattccaaca tggatgctga tttatatggg tataaatggg ctcgcgataa tgtcgggcaa
5281 tcaggtgcga caatctatcg cttgtatggg aagcccgatg cgccagagtt gtttctgaaa
5341 catggcaaag gtagcgttgc caatgatgtt acagatgaga tggtcagact aaactggctg
5401 acggaattta tgcctcttcc gaccatcaag cattttatcc gtactcctga tgatgcatgg
5461 ttactcacca ctgcgatccc cggaaaaaca gcattccagg tattagaaga atatcctgat
5521 tcaggtgaaa atattgttga tgcgctggca gtgttcctgc gccggttgca ttcgattcct
5581 gtttgtaatt gtccttttaa cagcgatcgc gtatttcgtc tcgctcaggc gcaatcacga
5641 atgaataacg gtttggttga tgcgagtgat tttgatgacg agcgtaatgg ctggcctgtt
5701 gaacaagtct ggaaagaaat gcataaactt ttgccattct caccggattc agtcgtcact

```
5761 catggtgatt tctcacttga taaccttatt tttgacgagg ggaaattaat aggttgtatt
5821 gatgttggac gagtcggaat cgcagaccga taccaggatc ttgccatcct atggaactgc
5881 ctcggtgagt tttctccttc attacagaaa cggctttttc aaaaatatgg tattgataat
5941 cctgatatga ataaattgca gtttcatttg atgctcgatg agtttttcta atcagaattg
6001 gttaattggt tgtaacactg gcagagcatt acgctgactt gacgggacgg cgcaagctca
6061 tgaccaaaat cccttaacgt gagttacgcg tcgttccact gagcgtcaga ccccgtagaa
6121 aagatcaaag gatcttcttg agatcctttt tttctgcgcg taatctgctg cttgcaaaca
6181 aaaaaaccac cgctaccagc ggtggtttgt ttgccggatc aagagctacc aactcttttt
6241 ccgaaggtaa ctggcttcag cagagcgcag ataccaaata ctgttcttct agtgtagccg
6301 tagttaggcc accacttcaa gaactctgta gcaccgccta catacctcgc tctgctaatc
6361 ctgttaccag tggctgctgc cagtggcgat aagtcgtgtc ttaccgggtt ggactcaaga
6421 cgatagttac cggataaggc gcagcggtcg ggctgaacgg ggggttcgtg cacacagccc
6481 agcttggagc gaacgaccta caccgaactg agatacctac agcgtgagct atgagaaagc
6541 gccacgcttc ccgaagggag aaaggcggac aggtatccgg taagcggcag ggtcggaaca
6601 ggagagcgca cgagggagct tccaggggga aacgcctggt atctttatag tcctgtcggg
6661 tttcgccacc tctgacttga gcgtcgattt ttgtgatgct cgtcaggggg gcggagccta
6721 tggaaaaacg ccagcaacgc ggccttttta cggttcctgg ccttttgctg gccttttgct
6781 cacatgtt
//
```

```
LOCUS       Exported                6160 bp ds-DNA    circular SYN 24-MAY-2019
DEFINITION  .
ACCESSION   .
VERSION     .
KEYWORDS    pDSAG-AgVg-aga-miR-14 sponges
SOURCE      synthetic DNA construct
  ORGANISM  synthetic DNA construct
REFERENCE   1  (bases 1 to 6160)
  AUTHORS   S Dong
  TITLE     Direct Submission
  JOURNAL   Exported Friday, May 24, 2019 from SnapGene 2.3.2
            http://www.snapgene.com
FEATURES             Location/Qualifiers
     source          1..6160
                     /organism="synthetic DNA construct"
                     /mol_type="other DNA"
     source          2171..2406
                     /organism="synthetic DNA construct"
                     /mol_type="other DNA"
     primer_bind     271..287
                     /note="M13 fwd"
                     /note="common sequencing primer, one of multiple similar
                     variants"
     protein_bind    complement(337..461)
                     /gene="mutant version of attR"
                     /bound_moiety="LR Clonase(TM)"
                     /note="attR4"
                     /note="recombination site for the Gateway(R) LR reaction"
     misc_feature    472..2170
                     /note="AgVg promoter"
     misc_feature    2171..2406
                     /note="aga-miR-14 sponges"
     polyA_signal    2519..2601
                     /note="SV40 poly(A) signal"
                     /note="SV40 polyadenylation signal"
     misc_feature    2639..2915
                     /note="attB"
     CDS             3249..3968
                     /codon_start=1
                     /product="enhanced GFP"
                     /note="EGFP"
                     /note="mammalian codon-optimized"
                     /translation="MVSKGEELFTGVVPILVELDGDVNGHKFSVSGEGEGDATYGKLTL
                     KFICTTGKLPVPWPTLVTTLTYGVQCFSRYPDHMKQHDFFKSAMPEGYVQERTIFFKDD
                     GNYKTRAEVKFEGDTLVNRIELKGIDFKEDGNILGHKLEYNYNSHNVYIMADKQKNGIK
                     VNFKIRHNIEDGSVQLADHYQQNTPIGDGPVLLPDNHYLSTQSALSKDPNEKRDHMVLL
                     EFVTAAGITLGMDELYK"
     polyA_signal    4090..4211
                     /note="SV40 poly(A) signal"
                     /note="SV40 polyadenylation signal"
```

```
     protein_bind    complement(4272..4371)
                     /gene="mutant version of attL"
                     /bound_moiety="LR Clonase(TM)"
                     /note="attL2"
                     /note="recombination site for the Gateway(R) LR reaction"
     promoter        complement(4402..4420)
                     /note="T7 promoter"
                     /note="promoter for bacteriophage T7 RNA polymerase"
     primer_bind     complement(4425..4441)
                     /note="M13 rev"
                     /note="common sequencing primer, one of multiple similar
                     variants"
     CDS             4554..5363
                     /codon_start=1
                     /gene="aph(3')-Ia"
                     /product="aminoglycoside phosphotransferase"
                     /note="KanR"
                     /note="confers resistance to kanamycin in bacteria or G418
                     (Geneticin(R)) in eukaryotes"
                     /translation="MSHIQRETSRPRLNSNMDADLYGYKWARDNVGQSGATIYRLYGKP
                     DAPELFLKHGKGSVANDVTDEMVRLNWLTEFMPLPTIKHFIRTPDDAWLLTTAIPGKTA
                     FQVLEEYPDSGENIVDALAVFLRRLHSIPVCNCPFNSDRVFRLAQAQSRMNNGLVDASD
                     FDDERNGWPVEQVWKEMHKLLPFSPDSVVTHGDFSLDNLIFDEGKLIGCIDVGRVGIAD
                     RYQDLAILWNCLGEFSPSLQKRLFQKYGIDNPDMNKLQFHLMLDEFF"
     rep_origin      5510..6098
                     /direction=RIGHT
                     /note="ori"
                     /note="high-copy-number ColE1/pMB1/pBR322/pUC origin of
                     replication"
ORIGIN
        1 ctttcctgcg ttatcccctg attctgtgga taaccgtatt accgcctttg agtgagctga
       61 taccgctcgc cgcagccgaa cgaccgagcg cagcgagtca gtgagcgagg aagcggaaga
      121 gcgcccaata cgcaaaccgc ctctccccgc gcgttggccg attcattaat gcagctggca
      181 cgacaggttt cccgactgga aagcgggcag tgagcgcaac gcaattaata cgcgtaccgc
      241 tagcatggat gttttcccag tcacgacgtt gtaaaacgac ggccagtctt aagctcgggc
      301 ccctacaggt cactaatacc atctaagtag ttgattcata gtgactggat atgttgtgtt
      361 ttacagtatt atgtagtctg tttttttatgc aaaatctaat ttaatatatt gatatttata
      421 tcattttacg tttctcgttc aacttttcta tacaaagttg gtaccggatc ctcgagttca
      481 actcgaccat aataattgat ccgtcaatcc atattggtcc gcaataatga aagttgcaag
      541 agtacgacgg tatgaaaaga ggttcagtaa gttgtaaact aatagtttct tcccaacgtt
      601 caaatgctgg caaatctttt cgcgggccgc acttcgtcga tcgctagtct taatgataat
      661 ttctgagaaa aaggtgctac tgcatctact atattctact ggatataaat gaaataacaa
      721 cgtgagactc acctacaaca tgtaatttat tgatggttta gtttaaccaa cctatgaaat
      781 aatttgatat agaaatttgt agtcgttttt ctatgaagta aaattctaaa atcaaacatt
      841 aaactgtttt gtagtacccg gactcatggt atggcttcta ttagccgtaa acaaagattt
      901 acaattgact aaggttaggt ccgacactgt aggagccagc gcgtcctttc aatacatcaa
      961 cggaccatct cgtgttgtta aatacttatt attattatgg tttgctaatt gatatgttcc
     1021 aagaccgatt tggatttcga aataagtatt ctctgattca ttttgggagc cggtctcgtg
     1081 atacagtcgt cagcccgtac gacttaacta cattctcgtc atgggttcaa gccccagatg
     1141 gaccgtgccg ccatacgtag agtcagtcct atcctgttat ggggggtaat acataagaca
```

1201 ctgaaagcca accccacaag tggtacagac aagccttgac cgacaattgt tgttgagcca
1261 aacagaagaa gaatccattt cgggaaatga ttttatcatt caatcaaacc agtcaatcat
1321 aaacatcata gttttaaata ctcaaaacta gttgagatct ttaaaacaca ttattttagt
1381 ttaattaaat gatctgttag ctagaaggta gatacgatat tttagacatt tcgtaataga
1441 tcgcaaatct ctattatgtt ggtaattcac ttcgtaaaac tcttaggcaa aactcttatt
1501 agtaaacaaa atactaatca aacactgata aactaacgcg atttatacat tggacaaaga
1561 agaggctgat ttttaaaaata ctcgctttaa aatttgcttc attcatcaat gtattgtaaa
1621 gcacataaag aacacaatca ttgactgaaa acaattccac gtctcagcca acttccagga
1681 tcaatgaaat agcaagttcc aagttccatt tcattgatta tggtaactac tgattatttt
1741 caataacaaa tacttcgaag actgcacaat tcaaaagtat gccagaaaga aaggattact
1801 atcaattgtg ggttaatcaa actaagacag gtggcaaaaa tggaaccatt gattaaggca
1861 gccactgacc gatttcatta aaaaatacac tcttggaagt ttccacacaa tctcagcttt
1921 tgccaatttt agcaaagacg ttgtgctgca ctgataagaa tcgaactgta aacatgtggg
1981 cagtaaaaat tatttcatcg ttcaacacgg cggtcattac actagtcgaa gcagctgaaa
2041 agatttgatg atagcaggac cgtgagatca gcaaatttga ggtataaaag atgatcctgc
2101 gaccaccaga aggcacattc gagctttgga gtgcattcaa agcatccggg caactgcgaa
2161 caaccgaacc taggagagct caaagactga cgcgtaggag agctcaaaga ctgagccata
2221 ggagagctca aagactgagc gataggagag ctcaaagact gagcgttagg agagctcaaa
2281 gactgagcaa taggagagct caaagactga gctttaggag agctcaaaga ctgaggcata
2341 ggagagctca aagactgagg cttaggagag ctcaaagact gagccatagg agagctcaaa
2401 gactgagctt ctagacataa tcagccatac cacatttgta gaggttttac ttgctttaaa
2461 aaacctccca cacctccccc tgaacctgaa acataaaatg aatgcaattg ttgttgttaa
2521 cttgtttatt gcagcttata atggttacaa ataaagcaat agcatcacaa atttcacaaa
2581 taaagcattt ttcttcactg cattctagtt gtggtttgtc caaactcatc aatgtatctc
2641 gacgatgtag gtcacagtct cgaagccgcg gtgcgggtgc cagggcgtgc ccttgggctc
2701 cccgggcgcg tactccacct cacccatctg gtccatcatg atgaacgggt cgaggtggcg
2761 gtagttgatc ccggcgaacg cgcggcgcac cgggaagccc tcgccctcga aaccgctggg
2821 cgcggtggtc acggtgagca cgggacgtgc gacggcgtcg gcgggtgcgg atacgcgggg
2881 cagcgtcagc gggttctcga cggtcacggc gggcaattcc tgcagacttc cggtatctcg
2941 cgtttgtttg atcgcacggt tcccacaatg gttaattcga gctcgcccgg ggatctaatt
3001 caattagaga ctaattcaat tagagctaat tcaattagga tccaagctta tcgatttcga
3061 accctcgacc gccggagtat aaatagaggc gcttcgtcta cggagcgaca attcaattca
3121 aacaagcaaa gtgaacacgt cgctaagcga aagctaagca aataaacaag cgcagctgaa
3181 caagctaaac aatcggggta ccgctagagt cgacggtacc gcgggcccgg gatccaccgg
3241 tcgccaccat ggtgagcaag ggcgaggagc tgttcaccgg ggtggtgccc atcctggtcg
3301 agctggacgg cgacgtaaac ggccacaagt tcagcgtgtc cggcgagggc gagggcgatg
3361 ccacctacgg caagctgacc ctgaagttca tctgcaccac cggcaagctg cccgtgccct
3421 ggcccaccct cgtgaccacc ctgacctacg gcgtgcagtg cttcagccgc taccccgacc
3481 acatgaagca gcacgacttc ttcaagtccg ccatgcccga aggctacgtc caggagcgca
3541 ccatcttctt caaggacgac ggcaactaca agacccgcgc cgaggtgaag ttcgagggcg
3601 acaccctggt gaaccgcatc gagctgaagg gcatcgactt caaggaggac ggcaacatcc
3661 tggggcacaa gctggagtac aactacaaca gccacaacgt ctatatcatg gccgacaagc
3721 agaagaacgg catcaaggtg aacttcaaga tccgccacaa catcgaggac ggcagcgtgc
3781 agctcgccga ccactaccag cagaacaccc ccatcggcga cggccccgtg ctgctgcccg
3841 acaaccacta cctgagcacc cagtccgccc tgagcaaaga ccccaacgag aagcgcgatc
3901 acatggtcct gctggagttc gtgaccgccg ccgggatcac tctcggcatg gacgagctgt
3961 acaagtaaag cggccgcgac tctagatcaa atcagccata ccacatttgt agaggtttta
4021 cttgctttaa aaaacctccc acacctcccc ctgaacctga aacataaaat gaatgcaatt
4081 gttgttgtta acttgtttat tgcagcttat aatggttaca aataaagcaa tagcatcaca
4141 aatttcacaa ataaagcatt tttttcactg cattctagtt gtggtttgtc caaactcatc

4201 aatgtatctt aaagcttatc gatacgcgta cggcgcgcct agagcggccg ccaccgcggt
4261 ggagctcgag tacccagctt tcttgtacaa agttggcatt ataagaaagc attgcttatc
4321 aatttgttgc aacgaacagg tcactatcag tcaaaataaa atcattattt gccatccagc
4381 tgcagggcgg ccgcgatatc ccctatagtg agtcgtatta catggtcata gctgtttcct
4441 ggcagctctg gcccgtgtct caaaatctct gatgttacat tgcacaagat aaaaatatat
4501 catcatgaac aataaaactg tctgcttaca taaacagtaa tacaaggggt gttatgagcc
4561 atattcaacg ggaaacgtcg aggccgcgat taaattccaa catggatgct gatttatatg
4621 ggtataaatg ggctcgcgat aatgtcgggc aatcaggtgc gacaatctat cgcttgtatg
4681 ggaagcccga tgcgccagag ttgtttctga aacatggcaa aggtagcgtt gccaatgatg
4741 ttacagatga gatggtcaga ctaaactggc tgacggaatt tatgcctctt ccgaccatca
4801 agcattttat ccgtactcct gatgatgcat ggttactcac cactgcgatc cccggaaaaa
4861 cagcattcca ggtattagaa gaatatcctg attcaggtga aaatattgtt gatgcgctgg
4921 cagtgttcct gcgccggttg cattcgattc ctgtttgtaa ttgtcctttt aacagcgatc
4981 gcgtatttcg tctcgctcag gcgcaatcac gaatgaataa cggtttggtt gatgcgagtg
5041 attttgatga cgagcgtaat ggctggcctg ttgaacaagt ctggaaagaa atgcataaac
5101 ttttgccatt ctcaccggat tcagtcgtca ctcatggtga tttctcactt gataacctta
5161 tttttgacga ggggaaatta ataggttgta ttgatgttgg acgagtcgga atcgcagacc
5221 gataccagga tcttgccatc ctatggaact gcctcggtga gttttctcct tcattacaga
5281 aacggctttt tcaaaaatat ggtattgata atcctgatat gaataaattg cagtttcatt
5341 tgatgctcga tgagtttttc taatcagaat tggttaattg gttgtaacac tggcagagca
5401 ttacgctgac ttgacgggac ggcgcaagct catgaccaaa atcccttaac gtgagttacg
5461 cgtcgttcca ctgagcgtca gaccccgtag aaaagatcaa aggatcttct tgagatcctt
5521 tttttctgcg cgtaatctgc tgcttgcaaa caaaaaaacc accgctacca gcggtggttt
5581 gtttgccgga tcaagagcta ccaactcttt ttccgaaggt aactggcttc agcagagcgc
5641 agataccaaa tactgttctt ctagtgtagc cgtagttagg ccaccacttc aagaactctg
5701 tagcaccgcc tacatacctc gctctgctaa tcctgttacc agtggctgct gccagtggcg
5761 ataagtcgtg tcttaccggg ttggactcaa gacgatagtt accggataag gcgcagcggt
5821 cgggctgaac ggggggttcg tgcacacagc ccagcttgga gcgaacgacc tacaccgaac
5881 tgagatacct acagcgtgag ctatgagaaa gcgccacgct tcccgaaggg agaaaggcgg
5941 acaggtatcc ggtaagcggc agggtcggaa caggagagcg cacgagggag cttccagggg
6001 gaaacgcctg gtatctttat agtcctgtcg ggtttcgcca cctctgactt gagcgtcgat
6061 ttttgtgatg ctcgtcaggg gggcggagcc tatggaaaaa cgccagcaac gcggcctttt
6121 tacggttcct ggccttttgc tggccttttg ctcacatgtt
//

```
LOCUS       Exported                6809 bp ds-DNA    circular SYN 24-MAY-2019
DEFINITION  .
ACCESSION   .
VERSION     .
KEYWORDS    pDSAT-AgCp-aga-miR-305 sponges
SOURCE      synthetic DNA construct
  ORGANISM  synthetic DNA construct
REFERENCE   1  (bases 1 to 6809)
  AUTHORS   .
  TITLE     Direct Submission
  JOURNAL   Exported Friday, May 24, 2019 from SnapGene 2.3.2
            http://www.snapgene.com
FEATURES             Location/Qualifiers
     source          1..6809
                     /organism="synthetic DNA construct"
                     /mol_type="other DNA"
     misc_feature    271..286
                     /note="M13F"
     misc_feature    472..2794
                     /note="AgCp promoter"
     misc_feature    2795..3051
                     /note="aga-miR-305 sponges"
     misc_feature    3057..3283
                     /note="SV40 term"
     misc_feature    3284..3560
                     /note="attB"
     misc_feature    3634..3683
                     /note="3x Pax6 binding sites"
     misc_feature    3723..3730
                     /note="TATA"
     misc_feature    3894..4610
                     /note="mTurquoise2"
     misc_feature    4639..4860
                     /note="SV40 term"
     misc_feature    complement(5070..5088)
                     /note="M13R"
     misc_feature    complement(5114..5132)
                     /note="DONR-RP"
ORIGIN
        1 ctttcctgcg ttatcccctg attctgtgga taaccgtatt accgcctttg agtgagctga
       61 taccgctcgc cgcagccgaa cgaccgagcg cagcgagtca gtgagcgagg aagcggaaga
      121 gcgcccaata cgcaaaccgc ctctccccgc gcgttggccg attcattaat gcagctggca
      181 cgacaggttt cccgactgga aagcgggcag tgagcgcaac gcaattaata cgcgtaccgc
      241 tagcatggat gttttcccag tcacgacgtt gtaaaacgac ggccagtctt aagctcgggc
      301 ccctacaggt cactaatacc atctaagtag ttgattcata gtgactggat atgttgtgtt
      361 ttacagtatt atgtagtctg ttttttatgc aaaatctaat ttaatatatt gatatttata
      421 tcattttacg tttctcgttc aacttttcta tacaaagttg gtaccggatc caggaggcat
      481 gtcataagcc actgatgcgc ttgacaacct atgactgatt aggtctatac gcggctagaa
      541 ccgacgttgc tttgatatac gtgttgatga aaaatgcgaa tagaacctgt caagactcgg
      601 atctgatcaa tcgctcttgc tcattcctat attatttctt cttcttcttg tggctcaaca
```

```
 661 accgttgccg gtcaaggcct gtaccctttc aatgactttt ggattacccc tcatagcagg
 721 atagttggtc ctatgtatgg cggcacggtc tatttggagc ttgaacccat gacgggctta
 781 ttgttaagtc gtacgagttg acatctgtat cataagacca gctatttatc ctatattatt
 841 tacaacatgt ttaagttatg gacctgtgct ttcaatgatg tttttttttt tatttattat
 901 ttcaactatt tcctaataca ttcgtgacac gtatgtaatg atccaaacaa tatcttggct
 961 aaagcagatc gtgtcctgtt gctttgaggg acctcaataa cctacaacat tacgtgtaca
1021 tgcggtcccc gagatacacg gtacctctta tacgcggatt cggagatacg cggttttcta
1081 aatttgacaa ttctttgagc aaattgtact gatttgacac atcaatttca aattgccaaa
1141 taatttccgt tttgatcgaa tgttaaaaac tatttcaaaa ggtttaaaac agttattttc
1201 agtcagaatc atatcaaata attcataaag tgactaaaac cgcccctac ttgcaaaatt
1261 acacaaaaat ttgtgatatt ttagcaggaa atcacgagat tcgacttacg cggaaattcg
1321 agatacgcgg tattttgcgg ccgttttcgg tccccattaa ccgtgtattt cgggaaccgc
1381 ctgtatatgc aagattttga gatttattca ccagctaatt ggggtggtta actttttcta
1441 caataagaga tatgatctaa ttcttttctt ataagagtcg tcgtatctac caatagctca
1501 caggtccttc caaatggaac cattaatatt atgtcaaaat cgttcaaaat tcaatgaaaa
1561 cgtgtaacga ttgaatatgt gaacagcatc gatatcccct taatgcttct cgttattctt
1621 tcaaccattc tttacatcct attggataag ttatgataga ttctcgtatt gtgtcgaata
1681 ttgatttctt cttatattta cgatatgaga ttgtttgagg acgatttaga atgagccgat
1741 ttgttgttaa atacatgact gaggaccaaa ttccttatct attactgcca atccccaacc
1801 aaacgagaca ctctcatgta gcactttgtt gatcgggttt gatgcaaagc ttgaagcaag
1861 ttcttgatag aaattaatac aaacgcaaat aacttcaact ataagcactg tatcatcgca
1921 ctctgtcatc ttcggtaaag ccaataaacc gcctgctaaa aatatttacc cagacaatcg
1981 tacggaattc ttcgccctca agtatcacga ttaccacgga gagtgggtgc aagcagttct
2041 attttatgca ttccccact cgataggaaa agcccctaca gccgctctta catgctatat
2101 gaagtggagt ggaggcacac tttacgccgc ccgatacgca gctcggaagc agaactgttc
2161 tgtgccggtt tggcacaaac gatgaatccg ttatgcgtac tggcaattgc gatcgcggta
2221 ggttaatgaa cgagcaataa acattgaatt tatctctctc tccctcgatc cggggcggtg
2281 tgtaattagc attgcacttg cccatgctct tcctaccatg atgtctaagc ccttcgtcgc
2341 ctgcatctaa aatagatgcg cgttctgtac acgtgccttc ccgcgcatct cttgagtgca
2401 cttgagacct tcggggtccg gtcgattatc gattatcgga aattctccac aaagcgctca
2461 agaagtcgtg cactgggtta agctgggctc gttgtgagtt tcttcacgtt cttcccaatc
2521 ctcttcaagc gacacattac ttatcgatta tcatcaactc agctcagccc gcaaggccgt
2581 taaaccttgt gcgcgcgcgc gttttggccc gcatcgccaa aaggggtata aaaagcccac
2641 tccagcagga accacccgat acagttcgtg caagcaagcg gtcgattgaa catggtgcga
2701 ttaaacagtg cagtcggctc ccggtggtgg gccccagcga tggccatcct ggcggtggcg
2761 ctcagtgtcg aagcggccga ggcggatcca ctcacagagc acctcccaag tacaatcgcg
2821 cagagcacct cccaagtaca atgccacaga gcacctccca agtacaatgc gacagagcac
2881 ctcccaagta caatgcgttc agagcacctc ccaagtacaa tgcaacagag cacctcccaa
2941 gtacaatgct tcagagcacc tcccaagtac aatggcacag agcacctccc aagtacaatg
3001 gctcagagca cctcccaagt acaatgccac agagcacctc ccaagtacaa tgcttctaga
3061 cataatcagc cataccacat ttgtagaggt tttacttgct ttaaaaaacc tcccacacct
3121 ccccctgaac ctgaaacata aaatgaatgc aattgttgtt gttaacttgt ttattgcagc
3181 ttataatggt tacaaataaa gcaatagcat cacaaatttc acaaataaag catttttctt
3241 cactgcattc tagttgtggt ttgtccaaac tcatcaatgt atctcgacga tgtaggtcac
3301 agtctcgaag ccgcggtgcg ggtgccaggg cgtgcccttg ggctccccgg gcgcgtactc
3361 cacctcaccc atctggtcca tcatgatgaa cgggtcgagg tggcggtagt tgatcccggc
3421 gaacgcgcgg cgcaccggga agccctcgcc ctcgaaaccg ctgggcgcgg tggtcacggt
3481 gagcacggga cgtgcgacgg cgtcggcggg tgcggatacg cggggcagcg tcagcgggtt
3541 ctcgacggtc acggcgggca attcctgcag acttccggta tctcgcgttt gtttgatcgc
3601 acggttccca caatggttaa ttcgagctcg cccggggatc taattcaatt agagactaat
```

3661 tcaattagag ctaattcaat taggatccaa gcttatcgat ttcgaaccct cgaccgccgg
3721 agtataaata gaggcgcttc gtctacggag cgacaattca attcaaacaa gcaaagtgaa
3781 cacgtcgcta agcgaaagct aagcaaataa acaagcgcag ctgaacaagc taaacaatcg
3841 gggtaccgct agagtcgacg gtaccgcggg cccgggatcc accggtcgcc accatggtga
3901 gcaagggcga ggagctgttc accggggtgg tgcccatcct ggtcgagctg gacggcgacg
3961 taaacggcca caagttcagc gtgtccggcg agggcgaggg cgatgccacc tacggcaagc
4021 tgaccctgaa gttcatctgc accaccggca agctgcccgt gccctggccc accctcgtga
4081 ccaccctgtc ctggggcgtg cagtgcttcg cccgctaccc cgaccacatg aagcagcacg
4141 acttcttcaa gtccgccatg cccgaaggct acgtccagga gcgcaccatc ttcttcaagg
4201 acgacggcaa ctacaagacc cgcgccgagg tgaagttcga gggcgacacc ctggtgaacc
4261 gcatcgagct gaagggcatc gacttcaagg aggacggcaa catcctgggg cacaagctgg
4321 agtacaacta ctttagcgac aacgtctata tcaccgccga caagcagaag aacggcatca
4381 aggccaactt caagatccgc cacaacatcg aggacggcgg cgtgcagctc gccgaccact
4441 accagcagaa cacccccatc ggcgacggcc ccgtgctgct gcccgacaac cactacctga
4501 gcacccagtc caagctgagc aaagacccca acgagaagcg cgatcacatg gtcctgctgg
4561 agttcgtgac cgccgccggg atcactctcg gcatggacga gctgtacaag tccggatgat
4621 agatctgacg ggtgatcaaa tcagccatac cacatttgta gaggttttac ttgctttaaa
4681 aaacctccca cacctccccc tgaacctgaa acataaaatg aatgcaattg ttgttgttaa
4741 cttgtttatt gcagcttata atggttacaa ataaagcaat agcatcacaa atttcacaaa
4801 taaagcattt ttttcactgc attctagttg tggtttgtcc aaactcatca atgtatctta
4861 aagcttatcg atacgcgtac ggcgcgccta gagcggccgc caccgcggtg gagctcgagt
4921 acccagcttt cttgtacaaa gttggcatta taagaaagca ttgcttatca atttgttgca
4981 acgaacaggt cactatcagt caaaataaaa tcattatttg ccatccagct gcagggcggc
5041 cgcgatatcc cctatagtga gtcgtattac atggtcatag ctgtttcctg gcagctctgg
5101 cccgtgtctc aaaatctctg atgttacatt gcacaagata aaaatatatc atcatgaaca
5161 ataaaactgt ctgcttacat aaacagtaat acaaggggtg ttatgagcca tattcaacgg
5221 gaaacgtcga ggccgcgatt aaattccaac atggatgctg atttatatgg gtataaatgg
5281 gctcgcgata atgtcgggca atcaggtgcg acaatctatc gcttgtatgg gaagcccgat
5341 gcgccagagt tgtttctgaa acatggcaaa ggtagcgttg ccaatgatgt tacagatgag
5401 atggtcagac taaactggct gacggaattt atgcctcttc cgaccatcaa gcattttatc
5461 cgtactcctg atgatgcatg gttactcacc actgcgatcc ccggaaaaac agcattccag
5521 gtattagaag aatatcctga ttcaggtgaa aatattgttg atgcgctggc agtgttcctg
5581 cgccggttgc attcgattcc tgtttgtaat tgtcctttta acagcgatcg cgtatttcgt
5641 ctcgctcagg cgcaatcacg aatgaataac ggtttggttg atgcgagtga ttttgatgac
5701 gagcgtaatg gctggcctgt tgaacaagtc tggaaagaaa tgcataaact tttgccattc
5761 tcaccggatt cagtcgtcac tcatggtgat ttctcacttg ataaccttat ttttgacgag
5821 gggaaattaa taggttgtat tgatgttgga cgagtcggaa tcgcagaccg ataccaggat
5881 cttgccatcc tatggaactg cctcggtgag ttttctcctt cattacagaa acggcttttt
5941 caaaaatatg gtattgataa tcctgatatg aataaattgc agtttcattt gatgctcgat
6001 gagtttttct aatcagaatt ggttaattgg ttgtaacact ggcagagcat tacgctgact
6061 tgacgggacg gcgcaagctc atgaccaaaa tcccttaacg tgagttacgc gtcgttccac
6121 tgagcgtcag accccgtaga aaagatcaaa ggatcttctt gagatccttt ttttctgcgc
6181 gtaatctgct gcttgcaaac aaaaaaacca ccgctaccag cggtggtttg tttgccggat
6241 caagagctac caactctttt tccgaaggta actggcttca gcagagcgca gataccaaat
6301 actgttcttc tagtgtagcc gtagttaggc caccacttca agaactctgt agcaccgcct
6361 acatacctcg ctctgctaat cctgttacca gtggctgctg ccagtggcga taagtcgtgt
6421 cttaccgggt tggactcaag acgatagtta ccggataagg cgcagcggtc gggctgaacg
6481 gggggttcgt gcacacagcc cagcttggag cgaacgacct acaccgaact gagataccta
6541 cagcgtgagc tatgagaaag cgccacgctt cccgaaggga gaaaggcgga caggtatccg
6601 gtaagcggca gggtcggaac aggagagcgc acgagggagc ttccaggggg aaacgcctgg

```
6661 tatctttata gtcctgtcgg gtttcgccac ctctgacttg agcgtcgatt tttgtgatgc
6721 tcgtcagggg ggcggagcct atggaaaaac gccagcaacg cggccttttt acggttcctg
6781 gccttttgct ggccttttgc tcacatgtt
//
```

```
LOCUS       Exported                6181 bp ds-DNA    circular SYN 24-MAY-2019
DEFINITION  .
ACCESSION   .
VERSION     .
KEYWORDS    pDSAG-AgVg-aga-miR-305 sponges
SOURCE      synthetic DNA construct
  ORGANISM  synthetic DNA construct
REFERENCE   1  (bases 1 to 6181)
  AUTHORS   S Dong
  TITLE     Direct Submission
  JOURNAL   Exported Friday, May 24, 2019 from SnapGene 2.3.2
            http://www.snapgene.com
FEATURES             Location/Qualifiers
     source          1..6181
                     /organism="synthetic DNA construct"
                     /mol_type="other DNA"
     primer_bind     271..287
                     /note="M13 fwd"
                     /note="common sequencing primer, one of multiple similar
                     variants"
     protein_bind    complement(337..461)
                     /gene="mutant version of attR"
                     /bound_moiety="LR Clonase(TM)"
                     /note="attR4"
                     /note="recombination site for the Gateway(R) LR reaction"
     misc_feature    472..2170
                     /note="AgVg promoter"
     misc_feature    2171..2427
                     /note="aga-miR-305 sponges"
     polyA_signal    2540..2622
                     /note="SV40 poly(A) signal"
                     /note="SV40 polyadenylation signal"
     misc_feature    2660..2936
                     /note="attB"
     CDS             3270..3989
                     /codon_start=1
                     /product="enhanced GFP"
                     /note="EGFP"
                     /note="mammalian codon-optimized"
                     /translation="MVSKGEELFTGVVPILVELDGDVNGHKFSVSGEGEGDATYGKLTL
                     KFICTTGKLPVPWPTLVTTLTYGVQCFSRYPDHMKQHDFFKSAMPEGYVQERTIFFKDD
                     GNYKTRAEVKFEGDTLVNRIELKGIDFKEDGNILGHKLEYNYNSHNVYIMADKQKNGIK
                     VNFKIRHNIEDGSVQLADHYQQNTPIGDGPVLLPDNHYLSTQSALSKDPNEKRDHMVLL
                     EFVTAAGITLGMDELYK"
     polyA_signal    4111..4232
                     /note="SV40 poly(A) signal"
                     /note="SV40 polyadenylation signal"
     protein_bind    complement(4293..4392)
                     /gene="mutant version of attL"
                     /bound_moiety="LR Clonase(TM)"
```

```
                     /note="attL2"
                     /note="recombination site for the Gateway(R) LR reaction"
     promoter        complement(4423..4441)
                     /note="T7 promoter"
                     /note="promoter for bacteriophage T7 RNA polymerase"
     primer_bind     complement(4446..4462)
                     /note="M13 rev"
                     /note="common sequencing primer, one of multiple similar
                     variants"
     CDS             4575..5384
                     /codon_start=1
                     /gene="aph(3')-Ia"
                     /product="aminoglycoside phosphotransferase"
                     /note="KanR"
                     /note="confers resistance to kanamycin in bacteria or G418
                     (Geneticin(R)) in eukaryotes"
                     /translation="MSHIQRETSRPRLNSNMDADLYGYKWARDNVGQSGATIYRLYGKP
                     DAPELFLKHGKGSVANDVTDEMVRLNWLTEFMPLPTIKHFIRTPDDAWLLTTAIPGKTA
                     FQVLEEYPDSGENIVDALAVFLRRLHSIPVCNCPFNSDRVFRLAQAQSRMNNGLVDASD
                     FDDERNGWPVEQVWKEMHKLLPFSPDSVVTHGDFSLDNLIFDEGKLIGCIDVGRVGIAD
                     RYQDLAILWNCLGEFSPSLQKRLFQKYGIDNPDMNKLQFHLMLDEFF"
     rep_origin      5531..6119
                     /direction=RIGHT
                     /note="ori"
                     /note="high-copy-number ColE1/pMB1/pBR322/pUC origin of
                     replication"
ORIGIN
        1 ctttcctgcg ttatcccctg attctgtgga taaccgtatt accgcctttg agtgagctga
       61 taccgctcgc cgcagccgaa cgaccgagcg cagcgagtca gtgagcgagg aagcggaaga
      121 gcgcccaata cgcaaaccgc ctctccccgc gcgttggccg attcattaat gcagctggca
      181 cgacaggttt cccgactgga aagcgggcag tgagcgcaac gcaattaata cgcgtaccgc
      241 tagcatggat gttttcccag tcacgacgtt gtaaaacgac ggccagtctt aagctcgggc
      301 ccctacaggt cactaatacc atctaagtag ttgattcata gtgactggat atgttgtgtt
      361 ttacagtatt atgtagtctg tttttttatgc aaaatctaat ttaatatatt gatatttata
      421 tcattttacg tttctcgttc aacttttcta tacaaagttg gtaccggatc ctcgagttca
      481 actcgaccat aataattgat ccgtcaatcc atattggtcc gcaataatga aagttgcaag
      541 agtacgacgg tatgaaaaga ggttcagtaa gttgtaaact aatagtttct tcccaacgtt
      601 caaatgctgg caaatctttt cgcgggccgc acttcgtcga tcgctagtct taatgataat
      661 ttctgagaaa aaggtgctac tgcatctact atattctact ggatataaat gaaataacaa
      721 cgtgagactc acctacaaca tgtaatttat tgatggttta gtttaaccaa cctatgaaat
      781 aatttgatat agaaatttgt agtcgttttt ctatgaagta aaattctaaa atcaaacatt
      841 aaactgtttt gtagtacccg gactcatggt atggcttcta ttagccgtaa acaaagattt
      901 acaattgact aaggttaggt ccgacactgt aggagccagc gcgtcctttc aatacatcaa
      961 cggaccatct cgtgttgtta aatacttatt attattatgg tttgctaatt gatatgttcc
     1021 aagaccgatt tggatttcga aataagtatt ctctgattca ttttgggagc cggtctcgtg
     1081 atacagtcgt cagcccgtac gacttaacta cattctcgtc atgggttcaa gccccagatg
     1141 gaccgtgccg ccatacgtag agtcagtcct atcctgttat gggggtaat acataagaca
     1201 ctgaaagcca accccacaag tggtacagac aagccttgac cgacaattgt tgttgagcca
     1261 aacagaagaa gaatccattt cgggaaatga ttttatcatt caatcaaacc agtcaatcat
     1321 aaacatcata gttttaaata ctcaaaacta gttgagatct ttaaaacaca ttattttagt
```

1381 ttaattaaat gatctgttag ctagaaggta gatacgatat tttagacatt tcgtaataga
1441 tcgcaaatct ctattatgtt ggtaattcac ttcgtaaaac tcttaggcaa aactcttatt
1501 agtaaacaaa atactaatca aacactgata aactaacgcg atttatacat tggacaaaga
1561 agaggctgat tttaaaaata ctcgctttaa aatttgcttc attcatcaat gtattgtaaa
1621 gcacataaag aacacaatca ttgactgaaa acaattccac gtctcagcca acttccagga
1681 tcaatgaaat agcaagttcc aagttccatt tcattgatta tggtaactac tgattatttt
1741 caataacaaa tacttcgaag actgcacaat tcaaaagtat gccagaaaga aaggattact
1801 atcaattgtg ggttaatcaa actaagacag gtggcaaaaa tggaaccatt gattaaggca
1861 gccactgacc gatttcatta aaaaatacac tcttggaagt ttccacacaa tctcagcttt
1921 tgccaatttt agcaaagacg ttgtgctgca ctgataagaa tcgaactgta aacatgtggg
1981 cagtaaaaat tatttcatcg ttcaacacgg cggtcattac actagtcgaa gcagctgaaa
2041 agatttgatg atagcaggac cgtgagatca gcaaatttga ggtataaaag atgatcctgc
2101 gaccaccaga aggcacattc gagctttgga gtgcattcaa agcatccggg caactgcgaa
2161 caaccgaacc cagagcacct cccaagtaca atcgcgcaga gcacctccca agtacaatgc
2221 cacagagcac ctcccaagta caatgcgaca gagcacctcc caagtacaat gcgttcagag
2281 cacctcccaa gtacaatgca acagagcacc tcccaagtac aatgcttcag agcacctccc
2341 aagtacaatg gcacagagca cctcccaagt acaatggctc agagcacctc ccaagtacaa
2401 tgccacagag cacctcccaa gtacaatgct tctagacata atcagccata ccacatttgt
2461 agaggtttta cttgctttaa aaaacctccc acacctcccc ctgaacctga aacataaaat
2521 gaatgcaatt gttgttgtta acttgtttat tgcagcttat aatggttaca aataaagcaa
2581 tagcatcaca aatttcacaa ataaagcatt tttcttcact gcattctagt tgtggtttgt
2641 ccaaactcat caatgtatct cgacgatgta ggtcacagtc tcgaagccgc ggtgcgggtg
2701 ccagggcgtg cccttgggct ccccgggcgc gtactccacc tcacccatct ggtccatcat
2761 gatgaacggg tcgaggtggc ggtagttgat cccggcgaac gcgcggcgca ccgggaagcc
2821 ctcgccctcg aaaccgctgg gcgcggtggt cacggtgagc acgggacgtg cgacggcgtc
2881 ggcgggtgcg gatacgcggg gcagcgtcag cgggttctcg acggtcacgg cgggcaattc
2941 ctgcagactt ccggtatctc gcgtttgttt gatcgcacgg ttcccacaat ggttaattcg
3001 agctcgcccg gggatctaat tcaattagag actaattcaa ttagagctaa ttcaattagg
3061 atccaagctt atcgatttcg aaccctcgac cgccggagta taaatagagg cgcttcgtct
3121 acggagcgac aattcaattc aaacaagcaa agtgaacacg tcgctaagcg aaagctaagc
3181 aaataaacaa gcgcagctga acaagctaaa caatcggggt accgctagag tcgacggtac
3241 cgcgggcccg ggatccaccg gtcgccacca tggtgagcaa gggcgaggag ctgttcaccg
3301 gggtggtgcc catcctggtc gagctggacg gcgacgtaaa cggccacaag ttcagcgtgt
3361 ccggcgaggg cgagggcgat gccacctacg gcaagctgac cctgaagttc atctgcacca
3421 ccggcaagct gcccgtgccc tggcccaccc tcgtgaccac cctgacctac ggcgtgcagt
3481 gcttcagccg ctaccccgac cacatgaagc agcacgactt cttcaagtcc gccatgcccg
3541 aaggctacgt ccaggagcgc accatcttct tcaaggacga cggcaactac aagacccgcg
3601 ccgaggtgaa gttcgagggc gacaccctgg tgaaccgcat cgagctgaag ggcatcgact
3661 tcaaggagga cggcaacatc ctggggcaca agctggagta caactacaac agccacaacg
3721 tctatatcat ggccgacaag cagaagaacg gcatcaaggt gaacttcaag atccgccaca
3781 acatcgagga cggcagcgtg cagctcgccg accactacca gcagaacacc cccatcggcg
3841 acggccccgt gctgctgccc gacaaccact acctgagcac ccagtccgcc ctgagcaaag
3901 accccaacga gaagcgcgat cacatggtcc tgctggagtt cgtgaccgcc gccgggatca
3961 ctctcggcat ggacgagctg tacaagtaaa gcggccgcga ctctagatca aatcagccat
4021 accacatttg tagaggtttt acttgcttta aaaaacctcc cacacctccc cctgaacctg
4081 aaacataaaa tgaatgcaat tgttgttgtt aacttgttta ttgcagctta taatggttac
4141 aaataaagca atagcatcac aaatttcaca aataaagcat ttttttcact gcattctagt
4201 tgtggtttgt ccaaactcat caatgtatct taaagcttat cgatacgcgt acggcgcgcc
4261 tagagcggcc gccaccgcgg tggagctcga gtacccagct ttcttgtaca aagttggcat
4321 tataagaaag cattgcttat caatttgttg caacgaacag gtcactatca gtcaaaataa

```
4381 aatcattatt tgccatccag ctgcagggcg gccgcgatat cccctatagt gagtcgtatt
4441 acatggtcat agctgtttcc tggcagctct ggcccgtgtc tcaaaatctc tgatgttaca
4501 ttgcacaaga taaaaatata tcatcatgaa caataaaact gtctgcttac ataaacagta
4561 atacaagggg tgttatgagc catattcaac gggaaacgtc gaggccgcga ttaaattcca
4621 acatggatgc tgatttatat gggtataaat gggctcgcga taatgtcggg caatcaggtg
4681 cgacaatcta tcgcttgtat gggaagcccg atgcgccaga gttgtttctg aaacatggca
4741 aaggtagcgt tgccaatgat gttacagatg agatggtcag actaaactgg ctgacggaat
4801 ttatgcctct tccgaccatc aagcatttta tccgtactcc tgatgatgca tggttactca
4861 ccactgcgat ccccggaaaa acagcattcc aggtattaga agaatatcct gattcaggtg
4921 aaaatattgt tgatgcgctg gcagtgttcc tgcgccggtt gcattcgatt cctgtttgta
4981 attgtccttt taacagcgat cgcgtatttc gtctcgctca ggcgcaatca cgaatgaata
5041 acggtttggt tgatgcgagt gattttgatg acgagcgtaa tggctggcct gttgaacaag
5101 tctggaaaga aatgcataaa cttttgccat tctcaccgga ttcagtcgtc actcatggtg
5161 atttctcact tgataacctt atttttgacg aggggaaatt aataggttgt attgatgttg
5221 gacgagtcgg aatcgcagac cgataccagg atcttgccat cctatggaac tgcctcggtg
5281 agttttctcc ttcattacag aaacggcttt ttcaaaaata tggtattgat aatcctgata
5341 tgaataaatt gcagtttcat ttgatgctcg atgagttttt ctaatcagaa ttggttaatt
5401 ggttgtaaca ctggcagagc attacgctga cttgacggga cggcgcaagc tcatgaccaa
5461 aatcccttaa cgtgagttac gcgtcgttcc actgagcgtc agaccccgta gaaaagatca
5521 aaggatcttc ttgagatcct ttttttctgc gcgtaatctg ctgcttgcaa acaaaaaaac
5581 caccgctacc agcggtggtt tgtttgccgg atcaagagct accaactctt tttccgaagg
5641 taactggctt cagcagagcg cagataccaa atactgttct tctagtgtag ccgtagttag
5701 gccaccactt caagaactct gtagcaccgc ctacatacct cgctctgcta atcctgttac
5761 cagtggctgc tgccagtggc gataagtcgt gtcttaccgg gttggactca agacgatagt
5821 taccggataa ggcgcagcgg tcgggctgaa cggggggttc gtgcacacag cccagcttgg
5881 agcgaacgac ctacaccgaa ctgagatacc tacagcgtga gctatgagaa agcgccacgc
5941 ttcccgaagg gagaaaggcg gacaggtatc cggtaagcgg cagggtcgga acaggagagc
6001 gcacgaggga gcttccaggg ggaaacgcct ggtatcttta tagtcctgtc gggtttcgcc
6061 acctctgact tgagcgtcga tttttgtgat gctcgtcagg ggggcggagc ctatggaaaa
6121 acgccagcaa cgcggccttt ttacggttcc tggccttttg ctggcctttt gctcacatgt
6181 t
```
//

```
LOCUS       Exported                7101 bp ds-DNA    circular SYN 24-MAY-2019
DEFINITION  .
ACCESSION   .
VERSION     .
KEYWORDS    pDSAG-AgCp-aga-miR-8 sponges
SOURCE      synthetic DNA construct
  ORGANISM  synthetic DNA construct
REFERENCE   1  (bases 1 to 7101)
  AUTHORS   S Dong
  TITLE     Direct Submission
  JOURNAL   Exported Friday, May 24, 2019 from SnapGene 2.3.2
            http://www.snapgene.com
FEATURES             Location/Qualifiers
     source          1..7101
                     /organism="synthetic DNA construct"
                     /mol_type="other DNA"
     source          472..2794
                     /organism="synthetic DNA construct"
                     /mol_type="other DNA"
     primer_bind     271..287
                     /note="M13 fwd"
                     /note="common sequencing primer, one of multiple similar
                     variants"
     protein_bind    complement(337..461)
                     /gene="mutant version of attR"
                     /bound_moiety="LR Clonase(TM)"
                     /note="attR4"
                     /note="recombination site for the Gateway(R) LR reaction"
     misc_feature    472..2794
                     /note="AgCp promoter"
     misc_feature    2795..3347
                     /note="aga-miR-8 sponges"
     polyA_signal    3460..3542
                     /note="SV40 poly(A) signal"
                     /note="SV40 polyadenylation signal"
     misc_feature    3580..3856
                     /note="attB"
     CDS             4190..4909
                     /codon_start=1
                     /product="enhanced GFP"
                     /note="EGFP"
                     /note="mammalian codon-optimized"
                     /translation="MVSKGEELFTGVVPILVELDGDVNGHKFSVSGEGEGDATYGKLTL
                     KFICTTGKLPVPWPTLVTTLTYGVQCFSRYPDHMKQHDFFKSAMPEGYVQERTIFFKDD
                     GNYKTRAEVKFEGDTLVNRIELKGIDFKEDGNILGHKLEYNYNSHNVYIMADKQKNGIK
                     VNFKIRHNIEDGSVQLADHYQQNTPIGDGPVLLPDNHYLSTQSALSKDPNEKRDHMVLL
                     EFVTAAGITLGMDELYK"
     polyA_signal    5031..5152
                     /note="SV40 poly(A) signal"
                     /note="SV40 polyadenylation signal"
```

```
     protein_bind    complement(5213..5312)
                     /gene="mutant version of attL"
                     /bound_moiety="LR Clonase(TM)"
                     /note="attL2"
                     /note="recombination site for the Gateway(R) LR reaction"
     promoter        complement(5343..5361)
                     /note="T7 promoter"
                     /note="promoter for bacteriophage T7 RNA polymerase"
     primer_bind     complement(5366..5382)
                     /note="M13 rev"
                     /note="common sequencing primer, one of multiple similar
                     variants"
     CDS             5495..6304
                     /codon_start=1
                     /gene="aph(3')-Ia"
                     /product="aminoglycoside phosphotransferase"
                     /note="KanR"
                     /note="confers resistance to kanamycin in bacteria or G418
                     (Geneticin(R)) in eukaryotes"
                     /translation="MSHIQRETSRPRLNSNMDADLYGYKWARDNVGQSGATIYRLYGKP
                     DAPELFLKHGKGSVANDVTDEMVRLNWLTEFMPLPTIKHFIRTPDDAWLLTTAIPGKTA
                     FQVLEEYPDSGENIVDALAVFLRRLHSIPVCNCPFNSDRVFRLAQAQSRMNNGLVDASD
                     FDDERNGWPVEQVWKEMHKLLPFSPDSVVTHGDFSLDNLIFDEGKLIGCIDVGRVGIAD
                     RYQDLAILWNCLGEFSPSLQKRLFQKYGIDNPDMNKLQFHLMLDEFF"
     rep_origin      6451..7039
                     /direction=RIGHT
                     /note="ori"
                     /note="high-copy-number ColE1/pMB1/pBR322/pUC origin of
                     replication"
ORIGIN
        1 ctttcctgcg ttatcccctg attctgtgga taaccgtatt accgcctttg agtgagctga
       61 taccgctcgc cgcagccgaa cgaccgagcg cagcgagtca gtgagcgagg aagcggaaga
      121 gcgcccaata cgcaaaccgc ctctccccgc gcgttggccg attcattaat gcagctggca
      181 cgacaggttt cccgactgga aagcgggcag tgagcgcaac gcaattaata cgcgtaccgc
      241 tagcatggat gttttcccag tcacgacgtt gtaaaacgac ggccagtctt aagctcgggc
      301 ccctacaggt cactaatacc atctaagtag ttgattcata gtgactggat atgttgtgtt
      361 ttacagtatt atgtagtctg ttttttatgc aaaatctaat ttaatatatt gatatttata
      421 tcattttacg tttctcgttc aacttttcta tacaaagttg gtaccggatc caggaggcat
      481 gtcataagcc actgatgcgc ttgacaacct atgactgatt aggtctatac gcggctagaa
      541 ccgacgttgc tttgatatac gtgttgatga aaaatgcgaa tagaacctgt caagactcgg
      601 atctgatcaa tcgctcttgc tcattcctat attatttctt cttcttcttg tggctcaaca
      661 accgttgccg gtcaaggcct gtaccctttc aatgactttt ggattacccc tcatagcagg
      721 atagttggtc ctatgtatgg cggcacggtc tatttggagc ttgaacccat gacgggctta
      781 ttgttaagtc gtacgagttg acatctgtat cataagacca gctatttatc ctatattatt
      841 tacaacatgt ttaagttatg gacctgtgct ttcaatgatg tttttttttt tatttattat
      901 ttcaactatt tcctaataca ttcgtgacac gtatgtaatg atccaaacaa tatcttggct
      961 aaagcagatc gtgtcctgtt gctttgaggg acctcaataa cctacaacat tacgtgtaca
     1021 tgcggtcccc gagatacacg gtacctctta tacgcggatt cggagatacg cggttttcta
     1081 aatttgacaa ttctttgagc aaattgtact gatttgacac atcaatttca aattgccaaa
     1141 taatttccgt tttgatcgaa tgttaaaaac tatttcaaaa ggtttaaaac agttattttc
```

1201 agtcagaatc atatcaaata attcataaag tgactaaaac cgcccctac ttgcaaaatt
1261 acacaaaaat ttgtgatatt ttagcaggaa atcacgagat tcgacttacg cggaaattcg
1321 agatacgcgg tattttgcgg ccgttttcgg tccccattaa ccgtgtattt cgggaaccgc
1381 ctgtatatgc aagattttga gatttattca ccagctaatt ggggtggtta actttttcta
1441 caataagaga tatgatctaa ttcttttctt ataagagtcg tcgtatctac caatagctca
1501 caggtccttc caaatggaac cattaatatt atgtcaaaat cgttcaaaat tcaatgaaaa
1561 cgtgtaacga ttgaatatgt gaacagcatc gatatcccct taatgcttct cgttattctt
1621 tcaaccattc tttacatcct attggataag ttatgataga ttctcgtatt gtgtcgaata
1681 ttgatttctt cttatattta cgatatgaga ttgtttgagg acgatttaga atgagccgat
1741 ttgttgttaa atacatgact gaggaccaaa ttccttatct attactgcca atccccaacc
1801 aaacgagaca ctctcatgta gcactttgtt gatcgggttt gatgcaaagc ttgaagcaag
1861 ttcttgatag aaattaatac aaacgcaaat aacttcaact ataagcactg tatcatcgca
1921 ctctgtcatc ttcggtaaag ccaataaacc gcctgctaaa aatatttacc cagacaatcg
1981 tacggaattc ttcgccctca agtatcacga ttaccacgga gagtgggtgc aagcagttct
2041 attttatgca ttccccact cgataggaaa agcccctaca gccgctctta catgctatat
2101 gaagtggagt ggaggcacac tttacgccgc ccgatacgca gctcggaagc agaactgttc
2161 tgtgccggtt tggcacaaac gatgaatccg ttatgcgtac tggcaattgc gatcgcggta
2221 ggttaatgaa cgagcaataa acattgaatt tatctctctc tccctcgatc cggggcggtg
2281 tgtaattagc attgcacttg cccatgctct tcctaccatg atgtctaagc ccttcgtcgc
2341 ctgcatctaa aatagatgcg cgttctgtac acgtgccttc ccgcgcatct cttgagtgca
2401 cttgagacct tcggggtccg gtcgattatc gattatcgga aattctccac aaagcgctca
2461 agaagtcgtg cactgggtta agctgggctc gttgtgagtt tcttcacgtt cttcccaatc
2521 ctcttcaagc gacacattac ttatcgatta tcatcaactc agctcagccc gcaaggccgt
2581 taaaccttgt gcgcgcgcgc gttttggccc gcatcgccaa aaggggtata aaaagcccac
2641 tccagcagga accacccgat acagttcgtg caagcaagcg gtcgattgaa catggtgcga
2701 ttaaacagtg cagtcggctc ccggtggtgg gccccagcga tggccatcct ggcggtggcg
2761 ctcagtgtcg aagcggccga ggcggatcca ctcaatttag gtgacactat aggacatctt
2821 tacactcagt attagccaga catctttaca ctcagtatta gcgagacatc tttacactca
2881 gtattagcgt gacatcttta cactcagtat tagcaagaca tctttacact cagtattagc
2941 ttgacatctt tacactcagt attaggcaga catctttaca ctcagtatta ggctgacatc
3001 tttacactca gtattagata gacatcttta cactcagtat tagattgaca tctttacact
3061 cagtattagg ctgacatctt tacactcagt attagccaga catctttaca ctcagtatta
3121 gcgagacatc tttacactca gtattagcgt gacatcttta cactcagtat tagcaagaca
3181 tctttacact cagtattagc ttgacatctt tacactcagt attaggcaga catctttaca
3241 ctcagtatta ggctgacatc tttacactca gtattagata gacatcttta cactcagtat
3301 tagattgaca tctttacact cagtattacc tatagtgagt cgtattagct tctagacata
3361 atcagccata ccacatttgt agaggtttta cttgctttaa aaaacctccc acacctcccc
3421 ctgaacctga aacataaaat gaatgcaatt gttgttgtta acttgtttat tgcagcttat
3481 aatggttaca aataaagcaa tagcatcaca aatttcacaa ataaagcatt tttcttcact
3541 gcattctagt tgtggtttgt ccaaactcat caatgtatct cgacgatgta ggtcacagtc
3601 tcgaagccgc ggtgcgggtg ccagggcgtg cccttgggct ccccgggcgc gtactccacc
3661 tcacccatct ggtccatcat gatgaacggg tcgaggtggc ggtagttgat cccggcgaac
3721 gcgcggcgca ccgggaagcc ctcgccctcg aaaccgctgg gcgcggtggt cacggtgagc
3781 acgggacgtg cgacggcgtc ggcgggtgcg gatacgcggg gcagcgtcag cgggttctcg
3841 acggtcacgg cgggcaattc ctgcagactt ccggtatctc gcgtttgttt gatcgcacgg
3901 ttcccacaat ggttaattcg agctcgcccg gggatctaat tcaattagag actaattcaa
3961 ttagagctaa ttcaattagg atccaagctt atcgatttcg aaccctcgac cgccggagta
4021 taaatagagg cgcttcgtct acggagcgac aattcaattc aaacaagcaa agtgaacacg
4081 tcgctaagcg aaagctaagc aaataaacaa gcgcagctga acaagctaaa caatcggggt
4141 accgctagag tcgacggtac cgcgggcccg ggatccaccg gtcgccacca tggtgagcaa

4201 gggcgaggag ctgttcaccg gggtggtgcc catcctggtc gagctggacg gcgacgtaaa
4261 cggccacaag ttcagcgtgt ccggcgaggg cgagggcgat gccacctacg gcaagctgac
4321 cctgaagttc atctgcacca ccggcaagct gcccgtgccc tggcccaccc tcgtgaccac
4381 cctgacctac ggcgtgcagt gcttcagccg ctaccccgac cacatgaagc agcacgactt
4441 cttcaagtcc gccatgcccg aaggctacgt ccaggagcgc accatcttct tcaaggacga
4501 cggcaactac aagacccgcg ccgaggtgaa gttcgagggc gacaccctgg tgaaccgcat
4561 cgagctgaag ggcatcgact tcaaggagga cggcaacatc ctggggcaca agctggagta
4621 caactacaac agccacaacg tctatatcat ggccgacaag cagaagaacg gcatcaaggt
4681 gaacttcaag atccgccaca acatcgagga cggcagcgtg cagctcgccg accactacca
4741 gcagaacacc cccatcggcg acggccccgt gctgctgccc gacaaccact acctgagcac
4801 ccagtccgcc ctgagcaaag accccaacga gaagcgcgat cacatggtcc tgctggagtt
4861 cgtgaccgcc gccgggatca ctctcggcat ggacgagctg tacaagtaaa gcggccgcga
4921 ctctagatca aatcagccat accacatttg tagaggtttt acttgcttta aaaaacctcc
4981 cacacctccc cctgaacctg aaacataaaa tgaatgcaat tgttgttgtt aacttgttta
5041 ttgcagctta taatggttac aaataaagca atagcatcac aaatttcaca aataaagcat
5101 ttttttcact gcattctagt tgtggtttgt ccaaactcat caatgtatct taaagcttat
5161 cgatacgcgt acggcgcgcc tagagcggcc gccaccgcgg tggagctcga gtacccagct
5221 ttcttgtaca aagttggcat tataagaaag cattgcttat caatttgttg caacgaacag
5281 gtcactatca gtcaaaataa aatcattatt tgccatccag ctgcagggcg gccgcgatat
5341 cccctatagt gagtcgtatt acatggtcat agctgtttcc tggcagctct ggcccgtgtc
5401 tcaaaatctc tgatgttaca ttgcacaaga taaaaatata tcatcatgaa caataaaact
5461 gtctgcttac ataaacagta atacaagggg tgttatgagc catattcaac gggaaacgtc
5521 gaggccgcga ttaaattcca acatggatgc tgatttatat gggtataaat gggctcgcga
5581 taatgtcggg caatcaggtg cgacaatcta tcgcttgtat gggaagcccg atgcgccaga
5641 gttgtttctg aaacatggca aaggtagcgt tgccaatgat gttacagatg agatggtcag
5701 actaaactgg ctgacggaat ttatgcctct tccgaccatc aagcatttta tccgtactcc
5761 tgatgatgca tggttactca ccactgcgat ccccggaaaa acagcattcc aggtattaga
5821 agaatatcct gattcaggtg aaaatattgt tgatgcgctg gcagtgttcc tgcgccggtt
5881 gcattcgatt cctgtttgta attgtccttt taacagcgat cgcgtatttc gtctcgctca
5941 ggcgcaatca cgaatgaata acggtttggt tgatgcgagt gattttgatg acgagcgtaa
6001 tggctggcct gttgaacaag tctggaaaga aatgcataaa cttttgccat tctcaccgga
6061 ttcagtcgtc actcatggtg atttctcact tgataacctt atttttgacg aggggaaatt
6121 aataggttgt attgatgttg gacgagtcgg aatcgcagac cgataccagg atcttgccat
6181 cctatggaac tgcctcggtg agttttctcc ttcattacag aaacggcttt ttcaaaaata
6241 tggtattgat aatcctgata tgaataaatt gcagtttcat ttgatgctcg atgagttttt
6301 ctaatcagaa ttggttaatt ggttgtaaca ctggcagagc attacgctga cttgacggga
6361 cggcgcaagc tcatgaccaa aatcccttaa cgtgagttac gcgtcgttcc actgagcgtc
6421 agaccccgta gaaaagatca aaggatcttc ttgagatcct ttttttctgc gcgtaatctg
6481 ctgcttgcaa acaaaaaaac caccgctacc agcggtggtt tgtttgccgg atcaagagct
6541 accaactctt tttccgaagg taactggctt cagcagagcg cagataccaa atactgttct
6601 tctagtgtag ccgtagttag gccaccactt caagaactct gtagcaccgc ctacatacct
6661 cgctctgcta atcctgttac cagtggctgc tgccagtggc gataagtcgt gtcttaccgg
6721 gttggactca agacgatagt taccggataa ggcgcagcgg tcgggctgaa cggggggttc
6781 gtgcacacag cccagcttgg agcgaacgac ctacaccgaa ctgagatacc tacagcgtga
6841 gctatgagaa agcgccacgc ttcccgaagg gagaaaggcg gacaggtatc cggtaagcgg
6901 cagggtcgga acaggagagc gcacgaggga gcttccaggg ggaaacgcct ggtatcttta
6961 tagtcctgtc gggtttcgcc acctctgact tgagcgtcga tttttgtgat gctcgtcagg
7021 ggggcggagc ctatggaaaa acgccagcaa cgcggccttt ttacggttcc tggccttttg
7081 ctggcctttt gctcacatgt t

//

```
LOCUS       Exported                6485 bp ds-DNA    circular SYN 24-MAY-2019
DEFINITION  .
ACCESSION   .
VERSION     .
KEYWORDS    pDSAT-AgVg-aga-miR-8 sponges
SOURCE      synthetic DNA construct
  ORGANISM  synthetic DNA construct
REFERENCE   1  (bases 1 to 6485)
  AUTHORS   .
  TITLE     Direct Submission
  JOURNAL   Exported Friday, May 24, 2019 from SnapGene 2.3.2
            http://www.snapgene.com
FEATURES             Location/Qualifiers
     source          1..6485
                     /organism="synthetic DNA construct"
                     /mol_type="other DNA"
     source          472..2170
                     /organism="synthetic DNA construct"
                     /mol_type="other DNA"
     source          2175..2727
                     /organism="synthetic DNA construct"
                     /mol_type="other DNA"
     misc_feature    271..286
                     /note="M13F"
     misc_feature    472..2170
                     /note="AgVg promoter"
     misc_feature    2175..2727
                     /note="aga-miR-8 sponges"
     misc_feature    2733..2959
                     /note="SV40 term"
     misc_feature    2960..3236
                     /note="attB"
     misc_feature    3310..3359
                     /note="3x Pax6 binding sites"
     misc_feature    3399..3406
                     /note="TATA"
     misc_feature    3570..4286
                     /note="mTurquoise2"
     misc_feature    4315..4536
                     /note="SV40 term"
     promoter        complement(4727..4745)
                     /note="T7 promoter"
                     /note="promoter for bacteriophage T7 RNA polymerase"
     misc_feature    complement(4746..4764)
                     /note="M13R"
     misc_feature    complement(4790..4808)
                     /note="DONR-RP"
     CDS             4879..5688
                     /codon_start=1
                     /gene="aph(3')-Ia"
```

```
                     /product="aminoglycoside phosphotransferase"
                     /note="KanR"
                     /note="confers resistance to kanamycin in bacteria or G418
                     (Geneticin(R)) in eukaryotes"
                     /translation="MSHIQRETSRPRLNSNMDADLYGYKWARDNVGQSGATIYRLYGKP
                     DAPELFLKHGKGSVANDVTDEMVRLNWLTEFMPLPTIKHFIRTPDDAWLLTTAIPGKTA
                     FQVLEEYPDSGENIVDALAVFLRRLHSIPVCNCPFNSDRVFRLAQAQSRMNNGLVDASD
                     FDDERNGWPVEQVWKEMHKLLPFSPDSVVTHGDFSLDNLIFDEGKLIGCIDVGRVGIAD
                     RYQDLAILWNCLGEFSPSLQKRLFQKYGIDNPDMNKLQFHLMLDEFF"
ORIGIN
        1 ctttcctgcg ttatcccctg attctgtgga taaccgtatt accgcctttg agtgagctga
       61 taccgctcgc cgcagccgaa cgaccgagcg cagcgagtca gtgagcgagg aagcggaaga
      121 gcgcccaata cgcaaaccgc ctctccccgc gcgttggccg attcattaat gcagctggca
      181 cgacaggttt cccgactgga aagcgggcag tgagcgcaac gcaattaata cgcgtaccgc
      241 tagcatggat gttttcccag tcacgacgtt gtaaaacgac ggccagtctt aagctcgggc
      301 ccctacaggt cactaatacc atctaagtag ttgattcata gtgactggat atgttgtgtt
      361 ttacagtatt atgtagtctg tttttatgc aaaatctaat ttaatatatt gatatttata
      421 tcattttacg tttctcgttc aacttttcta tacaaagttg gtaccggatc ctcgagttca
      481 actcgaccat aataattgat ccgtcaatcc atattggtcc gcaataatga aagttgcaag
      541 agtacgacgg tatgaaaaga ggttcagtaa gttgtaaact aatagtttct tcccaacgtt
      601 caaatgctgg caaatctttt cgcgggccgc acttcgtcga tcgctagtct taatgataat
      661 ttctgagaaa aaggtgctac tgcatctact atattctact ggatataaat gaaataacaa
      721 cgtgagactc acctacaaca tgtaatttat tgatggttta gtttaaccaa cctatgaaat
      781 aatttgatat agaaatttgt agtcgttttt ctatgaagta aaattctaaa atcaaacatt
      841 aaactgtttt gtagtacccg gactcatggt atggcttcta ttagccgtaa acaaagattt
      901 acaattgact aaggttaggt ccgacactgt aggagccagc gcgtcctttc aatacatcaa
      961 cggaccatct cgtgttgtta aatacttatt attattatgg tttgctaatt gatatgttcc
     1021 aagaccgatt tggatttcga aataagtatt ctctgattca ttttgggagc cggtctcgtg
     1081 atacagtcgt cagcccgtac gacttaacta cattctcgtc atgggttcaa gccccagatg
     1141 gaccgtgccg ccatacgtag agtcagtcct atcctgttat ggggggtaat acataagaca
     1201 ctgaaagcca accccacaag tggtacagac aagccttgac cgacaattgt tgttgagcca
     1261 aacagaagaa gaatccattt cgggaaatga ttttatcatt caatcaaacc agtcaatcat
     1321 aaacatcata gttttaaata ctcaaaacta gttgagatct ttaaaacaca ttattttagt
     1381 ttaattaaat gatctgttag ctagaaggta gatacgatat tttagacatt tcgtaataga
     1441 tcgcaaatct ctattatgtt ggtaattcac ttcgtaaaac tcttaggcaa aactcttatt
     1501 agtaaacaaa atactaatca aacactgata aactaacgcg atttatacat tggacaaaga
     1561 agaggctgat tttaaaaata ctcgctttaa aatttgcttc attcatcaat gtattgtaaa
     1621 gcacataaag aacacaatca ttgactgaaa acaattccac gtctcagcca acttccagga
     1681 tcaatgaaat agcaagttcc aagttccatt tcattgatta tggtaactac tgattatttt
     1741 caataacaaa tacttcgaag actgcacaat tcaaaagtat gccagaaaga aaggattact
     1801 atcaattgtg ggttaatcaa actaagacag gtggcaaaaa tggaaccatt gattaaggca
     1861 gccactgacc gatttcatta aaaaatacac tcttggaagt ttccacacaa tctcagcttt
     1921 tgccaatttt agcaaagacg ttgtgctgca ctgataagaa tcgaactgta aacatgtggg
     1981 cagtaaaaat tatttcatcg ttcaacacgg cggtcattac actagtcgaa gcagctgaaa
     2041 agatttgatg atagcaggac cgtgagatca gcaaatttga ggtataaaag atgatcctgc
     2101 gaccaccaga aggcacattc gagctttgga gtgcattcaa agcatccggg caactgcgaa
     2161 caaccgaacc ctcaatttag gtgacactat aggacatctt tacactcagt attagccaga
     2221 catctttaca ctcagtatta gcgagacatc tttacactca gtattagcgt gacatcttta
     2281 cactcagtat tagcaagaca tctttacact cagtattagc ttgacatctt tacactcagt
     2341 attaggcaga catctttaca ctcagtatta ggctgacatc tttacactca gtattagata
```

2401 gacatcttta cactcagtat tagattgaca tctttacact cagtattagg ctgacatctt
2461 tacactcagt attagccaga catctttaca ctcagtatta gcgagacatc tttacactca
2521 gtattagcgt gacatcttta cactcagtat tagcaagaca tctttacact cagtattagc
2581 ttgacatctt tacactcagt attaggcaga catctttaca ctcagtatta ggctgacatc
2641 tttacactca gtattagata gacatcttta cactcagtat tagattgaca tctttacact
2701 cagtattacc tatagtgagt cgtattagct tctagacata atcagccata ccacatttgt
2761 agaggtttta cttgctttaa aaaacctccc acacctcccc ctgaacctga aacataaaat
2821 gaatgcaatt gttgttgtta acttgtttat tgcagcttat aatggttaca aataaagcaa
2881 tagcatcaca aatttcacaa ataaagcatt tttcttcact gcattctagt tgtggtttgt
2941 ccaaactcat caatgtatct cgacgatgta ggtcacagtc tcgaagccgc ggtgcgggtg
3001 ccagggcgtg cccttgggct ccccgggcgc gtactccacc tcacccatct ggtccatcat
3061 gatgaacggg tcgaggtggc ggtagttgat cccggcgaac gcgcggcgca ccgggaagcc
3121 ctcgccctcg aaaccgctgg gcgcggtggt cacggtgagc acgggacgtg cgacggcgtc
3181 ggcgggtgcg gatacgcggg gcagcgtcag cgggttctcg acggtcacgg cgggcaattc
3241 ctgcagactt ccggtatctc gcgtttgttt gatcgcacgg ttcccacaat ggttaattcg
3301 agctcgcccg gggatctaat tcaattagag actaattcaa ttagagctaa ttcaattagg
3361 atccaagctt atcgatttcg aaccctcgac cgccggagta taaatagagg cgcttcgtct
3421 acggagcgac aattcaattc aaacaagcaa agtgaacacg tcgctaagcg aaagctaagc
3481 aaataaacaa gcgcagctga acaagctaaa caatcggggt accgctagag tcgacggtac
3541 cgcgggcccg ggatccaccg gtcgccacca tggtgagcaa gggcgaggag ctgttcaccg
3601 gggtggtgcc catcctggtc gagctggacg gcgacgtaaa cggccacaag ttcagcgtgt
3661 ccggcgaggg cgagggcgat gccacctacg gcaagctgac cctgaagttc atctgcacca
3721 ccggcaagct gcccgtgccc tggcccaccc tcgtgaccac cctgtcctgg ggcgtgcagt
3781 gcttcgcccg ctaccccgac cacatgaagc agcacgactt cttcaagtcc gccatgcccg
3841 aaggctacgt ccaggagcgc accatcttct tcaaggacga cggcaactac aagacccgcg
3901 ccgaggtgaa gttcgagggc gacaccctgg tgaaccgcat cgagctgaag ggcatcgact
3961 tcaaggagga cggcaacatc ctggggcaca agctggagta caactacttt agcgacaacg
4021 tctatatcac cgccgacaag cagaagaacg gcatcaaggc caacttcaag atccgccaca
4081 acatcgagga cggcggcgtg cagctcgccg accactacca gcagaacacc cccatcggcg
4141 acggccccgt gctgctgccc gacaaccact acctgagcac ccagtccaag ctgagcaaag
4201 accccaacga gaagcgcgat cacatggtcc tgctggagtt cgtgaccgcc gccgggatca
4261 ctctcggcat ggacgagctg tacaagtccg gatgatagat ctgacgggtg atcaaatcag
4321 ccataccaca tttgtagagg ttttacttgc tttaaaaaac ctcccacacc tccccctgaa
4381 cctgaaacat aaaatgaatg caattgttgt tgttaacttg tttattgcag cttataatgg
4441 ttacaaataa agcaatagca tcacaaattt cacaaataaa gcattttttt cactgcattc
4501 tagttgtggt ttgtccaaac tcatcaatgt atcttaaagc ttatcgatac gcgtacggcg
4561 cgcctagagc ggccgccacc gcggtggagc tcgagtaccc agctttcttg tacaaagttg
4621 gcattataag aaagcattgc ttatcaattt gttgcaacga acaggtcact atcagtcaaa
4681 ataaaatcat tatttgccat ccagctgcag ggcggccgcg atatccccta tagtgagtcg
4741 tattacatgg tcatagctgt ttcctggcag ctctggcccg tgtctcaaaa tctctgatgt
4801 tacattgcac aagataaaaa tatatcatca tgaacaataa aactgtctgc ttacataaac
4861 agtaatacaa ggggtgttat gagccatatt caacgggaaa cgtcgaggcc gcgattaaat
4921 tccaacatgg atgctgattt atatgggtat aaatgggctc gcgataatgt cgggcaatca
4981 ggtgcgacaa tctatcgctt gtatgggaag cccgatgcgc cagagttgtt tctgaaacat
5041 ggcaaaggta gcgttgccaa tgatgttaca gatgagatgg tcagactaaa ctggctgacg
5101 gaatttatgc ctcttccgac catcaagcat tttatccgta ctcctgatga tgcatggtta
5161 ctcaccactg cgatccccgg aaaaacagca ttccaggtat tagaagaata tcctgattca
5221 ggtgaaaata ttgttgatgc gctggcagtg ttcctgcgcc ggttgcattc gattcctgtt
5281 tgtaattgtc cttttaacag cgatcgcgta tttcgtctcg ctcaggcgca atcacgaatg
5341 aataacggtt tggttgatgc gagtgatttt gatgacgagc gtaatggctg gcctgttgaa

```
5401 caagtctgga aagaaatgca taaacttttg ccattctcac cggattcagt cgtcactcat
5461 ggtgatttct cacttgataa ccttattttt gacgagggga aattaatagg ttgtattgat
5521 gttggacgag tcggaatcgc agaccgatac caggatcttg ccatcctatg gaactgcctc
5581 ggtgagtttt ctccttcatt acagaaacgg ctttttcaaa aatatggtat tgataatcct
5641 gatatgaata aattgcagtt tcatttgatg ctcgatgagt ttttctaatc agaattggtt
5701 aattggttgt aacactggca gagcattacg ctgacttgac gggacggcgc aagctcatga
5761 ccaaaatccc ttaacgtgag ttacgcgtcg ttccactgag cgtcagaccc cgtagaaaag
5821 atcaaaggat cttcttgaga tccttttttt ctgcgcgtaa tctgctgctt gcaaacaaaa
5881 aaaccaccgc taccagcggt ggtttgtttg ccggatcaag agctaccaac tctttttccg
5941 aaggtaactg gcttcagcag agcgcagata ccaaatactg ttcttctagt gtagccgtag
6001 ttaggccacc acttcaagaa ctctgtagca ccgcctacat acctcgctct gctaatcctg
6061 ttaccagtgg ctgctgccag tggcgataag tcgtgtctta ccgggttgga ctcaagacga
6121 tagttaccgg ataaggcgca gcggtcgggc tgaacggggg gttcgtgcac acagcccagc
6181 ttggagcgaa cgacctacac cgaactgaga tacctacagc gtgagctatg agaaagcgcc
6241 acgcttcccg aagggagaaa ggcggacagg tatccggtaa gcggcagggt cggaacagga
6301 gagcgcacga gggagcttcc agggggaaac gcctggtatc tttatagtcc tgtcgggttt
6361 cgccacctct gacttgagcg tcgatttttg tgatgctcgt caggggggcg gagcctatgg
6421 aaaaacgcca gcaacgcggc ctttttacgg ttcctggcct tttgctggcc ttttgctcac
6481 atgtt
//
```

**Primers and DNA sequences**

| Name | Sequence (5' to 3') | Purpose |
| --- | --- | --- |
| AgCp-BsaI-F | GTGGTCTCTATCCAGGAGGCATGTCATAAGCCAC | AgCp promoter cloning |
| AgCp-BsaI-R | GTGGTCTCTTGAGTGGATCCGCCTCGGCCGCTTC | |
| AgVg-BsaI-F1 | GTGGTCTCTATCCTCGAGTTCAACTCGACCATA | AgVg promoter cloning |
| AgCp-BsaI-R | GTGGTCTCTTGAGGGTTCGGTTGTTCGCAGTTGC | |
| miR-8 | TAATACTGTCAGGTAAAGATGTC | qPCR for miRNA expression |
| miR-14 | TCAGTCTTTTTCTCTCTCCTA | |
| miR-305 | ATTGTACTTCATCAGGTGCTCTG | |
| miR-14 sponges | GTGGTCTCTCTCATAGGAGAGCTCAAAGACTGACGCGTAGGAGAGCTCAAAGACTGAGCCATAGGAGAGCTCAAAGACTGAGCGATAGGAGAGCTCAAAGACTGAGCGTTAGGAGAGCTCAAAGACTGAGCAATAGGAGAGCTCAAAGACTGAGCTTTAGGAGAGCTCAAAGACTGAGGCATAGGAGAGCTCAAAGACTGAGGCTTAGGAGAGCTCAAAGACTGAGCCATAGGAGAGCTCAAAGACTGAGCTTAGAGACCAC | Golden gate assembly |
| miR-305 sponges | GTGGTCTCTCTCACAGAGCACCTCCCAAGTACAATCGCGCAGAGCACCTCCCAAGTACAATGCCACAGAGCACCTCCCAAGTACAATGCGACAGAGCACCTCCCAAGTACAATGCGTTCAGAGCACCTCCCAAGTACAATGCAACAGAGCACCTCCCAAGTACAATGCTTCAGAGCACCTCCCAAGTACAATGGCACAGAGCACCTCCCAAGTACAATGGCTCAGAGCACCTCCCAAGTACAATGCCACAGAGCACCTCCCAAGTACAATGCTTAGAGACCAC | Golden gate assembly |
| miR-8 sponges | GTGGTCTCTCTCAATTTAGGTGACACTATAGGACATCTTTACACTCAGTATTAGCCAGACATCTTTACACTCAGTATTAGCGAGACATCTTTACACTCAGTATTAGCGTGACATCTTTACACTCAGTATTAGCAAGACATCTTTACACTCAGTATTAGCTTGACATCTTTACACTCAGTATTAGGCAGACATCTTTACACTCAGTATTAGGCTGACATCTTTACACTCAGTATTAGATAGACATCTTTACACTCAGTATTAGATTGACATCTTTACACTCAGTATTAGGCTGACATCTTTACACTCAGTATTAGCCAGACATCTTTACACTCAGTATTAGCGAGACATCTTTACACTCAGTATTAGCGTGACATCTTTACACTCAGTATTAGCAAGACATCTTTACACTCAGTATTAGCTTGACATCTTTACACTCAGTATTAGGCAGACATCTTTACACTCAGTATTAGGCTGACATCTTTACACTCAGTATTAGATAGACATCTTTACACTCAGTATTAGATTGACATCTTTACACTCAGTATTACCTATAGTGAGTCGTATTAGCTTAGAGACCAC | Golden gate assembly |
